# Unravelling the devaluation puzzle: Empirical insights into the transmission channel on balance of payments and output in Ethiopia

**DOI:** 10.12688/f1000research.151984.1

**Published:** 2024-06-24

**Authors:** Yigermal Maru Ayinewa, Mesele Belay Zegeye, Tesfahun Ayanaw Alemu, Abate Belaye Tefera

**Affiliations:** 1 Economics, Woldia University, Weldiya, Amhara, Ethiopia

**Keywords:** Devaluation, Balance of Payments, Output growth, SVAR Model, Transmission Channels

## Abstract

**Background:**

Empirical studies on the impact of devaluation in developing countries, including Ethiopia, have revealed diverse and mixed results. The effects can be positive or negative depending on the specific economic context and policies in place.This study addresses the devaluation puzzle by providing a more comprehensive and nuanced understanding of how devaluation affects the balance of payments and output.

**Methods:**

To achieve this, we employ a recursive structural vector autoregressive (SVAR) model focusing on Ethiopia from 2001Q1 to 2023Q4.

**Results:**

The findings reveal that exchange rate shocks negatively affect the balance of payments in both the short and long run, with a greater impact observed in the short run. Shocks in other variables, such as foreign exchange rate reserves, inflation, and interest rates, exert a stronger influence on the balance of payments than the exchange rate itself in the long run. In addition, shocks in the exchange rate significantly impact foreign exchange rate reserves in the long run, highlighting the complex dynamics of exchange rates and the indirect nature of the exchange rate channel. Exchange rate shocks also negatively impact output growth in both the short and long run.

**Conclusion:**

The study concludes that devaluation has a contractionary effect through various channels. Devaluation increases the money supply, leading to inflationary pressures and a decline in output. It also increases interest rates, which further reduces output. In addition, devaluation reduces foreign exchange reserves, resulting in a fall in the balance of payments and a decrease in output. We suggest policymakers prioritize exchange rate stability through practical monetary and fiscal policies. In general, International financial institutions, particularly the National Bank of Ethiopia, should reconsider their devaluation-focused policies, considering the complexities and potential negative consequences associated with devaluation.

## 1. Introduction

This study examines the complex dynamics between devaluation, the balance of payments, and output in Ethiopia. Devaluation, a decline in the value of a country’s currency relative to foreign currency, is recognized as a tool for stabilizing the foreign sector of an economy. ^1^^–^^3^ During the 1970s and 1980s, many developing countries faced severe balance of payments deterioration due to overvalued currencies and high unemployment rates. ^4^ Consequently, these countries sought assistance from international organizations, such as the International Monetary Fund (IMF) and the World Bank (WB), both of which were cautious in providing aid without specific conditions, including the implementation of structural adjustment programs (SAPs). ^5^^,^^6^ As a result, numerous African countries, including Ethiopia, agreed to implement SAPs and fulfil the World Bank and IMF requirements.

The impact of exchange rate movements in developing countries varies depending on the nature of their principal exports. It is governed by the Marshall-Lerner condition, which determines the effect of exchange rate depreciation or appreciation on the balance of payment. ^7^

Since 1992, Ethiopia has followed a managed floating exchange rate system, which has resulted in significant fluctuations in the official exchange rate. One notable instance of devaluation occurred when the exchange rate surged from 2.07 birr/dollar to 5 birr/dollar, representing a remarkable devaluation rate of 142%, the highest in Ethiopian history. In September 2010, there was another significant increase in the exchange rate, with a rise of 16.7% from 13.6 birr/dollar to 16.3 birr/dollar. Additionally, in November 2017, the Ethiopian birr was officially devalued by 15% against the US dollar, causing the exchange rate to shift from 23.3 birr per dollar to 27 birr per dollar. To provide a broader perspective, it is important to highlight the overall trend in Ethiopia’s exchange rate. For example, in 1973, the exchange rate was 2.1 birr/dollar, and by 2024, it had increased to 56.559 birr/dollar on average, demonstrating an average annual growth rate of 7.4%. ^3^^,^^8^^–^^10^

Empirical studies conducted in developing countries in general, and in Ethiopia in particular, the impact of devaluation on the balance of payments, and output growth reveals diverse perspectives and mixed results, which is positive and negative effect. Studies such as, ^6^^,^^11^^–^^18^ devaluation affects positively the balance of payment trough improving trade balance, current account balance and output growth. However, several other studies ^19^^–^^24^ suggest that devaluation negatively affect trade balance in the short run and had no any significant gain in the long run, and output is negatively affected both in the short run and short run, contractionary impact. This contractionary impact is attributed to reduced aggregate demand, income redistribution from domestic to foreign sectors, and deterioration of the trade balance. ^25^ In addition, devaluation does not have a significant impact on Ethiopian economy because it increases the rate of growth of imports and decreased the rate of growth of export. ^26^

Hence, this study aims to make the following contributions to the existing literature: first, while previous studies have primarily focused on examining the individual effects of devaluation on trade balance, balance of payment, and output growth; (e.g., Refs. 3, 7, 11, 13, 16, 18– 21, 27– 32), it has been observed that there are dynamic relationships among devaluation, balance of payment, and output growth. Thus, this study seeks to provide empirical evidence on the dynamics of devaluation, the balance of payment, and output growth. Second, prior studies have primarily concentrated on illustrating the transmission mechanism of monetary policy while neglecting to investigate the transmission of devaluation on the balance of payments and output growth (e.g., Refs. 4, 33, 34). Thus, further investigation is needed to explore the complex channels through which devaluation influences the balance of payments and output growth. Third, many previous studies have revealed inconclusive results regarding the dynamics of devaluation, balance of payment, and output growth. Therefore, this study contributes by providing more critical insights into these dynamics. Finally, this study resolves the devaluation puzzle by offering a more comprehensive and nuanced understanding of the transmission channels through which devaluation impacts the balance of payments and output growth. In addition to the exchange rate, this study considers other crucial variables such as foreign asset reserves, interest rates, money supply, and inflation, thereby providing a more comprehensive picture of the transmission channel. This study employs a structural VAR model and uses a comprehensive dataset covering the period from 2001Q1 to 2023Q4 to enhance our understanding of the complex dynamics between devaluation, balance of payments, and output growth in the Ethiopian context.

The remaining section of the paper is structured as follows; Section 2 presents data description and methodology, Section 3 reports results and discusses the study’s results, and Section 4 provides the conclusion and policy recommendations of the study.

## 2. Methods

### 2.1 Data

This study uses quarterly data obtained from the National Bank of Ethiopia (NBE) to investigate the effects of devaluation on the balance of payments and output. The dataset comprises seven variables: exchange rate, balance of payments, money supply, inflation, interest rates, foreign asset reserves, and real gross domestic product (GDP). To ensure consistency, the real GDP data initially available only annually from the NBE are interpolated to quarterly data using the quadratic-sum approach of E-views 12. The dataset covers a period of 92 observations from 2001Q1 to 2023Q4. The use of quarterly data facilitates the identification of structural shocks, reduces the likelihood of structural breaks, and captures important intra-year dynamics. ^35^

### 2.2 Theoretical framework and model specification

In line with established economic theories and empirical literature, this study builds a model that enables exploration of the transmission channels through which the exchange rate affects the balance of payments and output. By employing rigorous econometric techniques and a sound theoretical framework, this study provides valuable insights into the dynamics of exchange rate transmission in relation to the Ethiopian economy.

The model specification begins with the identification and determination of independent variables based on economic theory. ^36^ In accordance with the theoretical framework and empirical evidence, models can be specified to capture the impact of devaluation on the balance of payments and output growth by introducing structural shocks. The theoretical model examines the transmission channels of devaluation in the balance of payments and output. The model incorporates both the demand and supply sides of the economy to analyse the effects of devaluation. ^37^ On the demand side, the model considers the national income identity equation and aggregate supply.

**The balance of payment channel:** devaluation can improve the current account of an economy in equilibrium if the combined elasticities of foreign demand for exports and domestic demand for imports exceed unity, known as the Marshall-Lerner condition. ^37^^–^^39^ This indicates that devaluation has both a price effect, making exports more competitive and imports more expensive, and a volume effect, leading to increased export volumes and decreased import volumes. Moreover, devaluation can impact the current account balance by influencing the marginal propensity to absorb, income levels, and direct absorption. Furthermore, the imbalance in the money market, as explained by the monetary approach, contributes to the disequilibrium in the balance of payments. In addition, shocks in the money supply, central bank’s foreign exchange reserves, inflation, domestic interest rates, and real output levels influence the balance of payments, and are specified in Eq. (1).

bop=f(e,i,ms,inf,fxre,rgdp) (1)

Where, *bop* - is balance of payment, *e* - is nominal exchange rate, $M_{s}$ - is money supply (M2), *inf* - is inflation, *I* - is domestic interest rate, *fxre* - is foreign asset reserve, and *rgdp* - is real GDP.

**The exchange rate channel: i**n a small open economy with free capital mobility, the domestic interest rate must equal the world interest rate in equilibrium. ^37^ Therefore, by equating the domestic interest rate with the world interest rate, we can determine the equilibrium level of the current account. However, in our economy, the interest rate is not determined through this mechanism but is instead fixed by the central bank. A higher domestic interest rate encourages domestic savings and reduces firms’ investment in physical capital. As a result, the domestic interest rate itself affects the current account by reducing exports. The relationship between the interest rate and the exchange rate, regardless of whether devaluation is expansionary or contractionary, is influenced by the type of shock. ^40^ This relationship between the domestic interest rate and investment demonstrates that the domestic interest rate has its own impact on the balance of payments.

This study also considers the inclusion of the price level in the balance of payment equation to account for the price environment of an economy. This is crucial in determining whether devaluation is inflationary or not, as inflation has a significant impact on output. ^41^ An inflationary environment can have negative effects on output. ^14^^,^^42^ This is because it can lead to inefficient allocation of resources due to distortions in relative prices and increased administrative costs for firms ^37^^,^^38^; clearly indicate the interaction between the balance of payments and the exchange rate. According to the elasticity approach of exchange rate determination, the exchange rate is determined by the flow of currency through the exchange rate market affecting trade balance, which affects the balance of payments component. In the portfolio view of exchange rate determination; the change in asset prices in the stock market through changes in the price of bonds and money affects the exchange rate.

Therefore, we include BOP in the exchange rate model. We have also understood that an increase in the money supply lowers interest rates, reduces borrowing costs, and promotes investment, which might enhance domestic output. In addition, a higher money supply will reduce the value of the currency. This relationship shows that money supply affects investment and output. ^40^ As in the quantity theory of money, the nominal exchange rate is determined by the quantity of money printed by the central banks that is money supply. This is seeing the exchange rate from the viewpoint of the monetary approach; interest rate and money supply influence exchange rate determination. There is a long run relationship between foreign reserves and exchange rate. ^43^ Moreover; any changes in foreign reserves would lead to fluctuations in the exchange rate but not vice versa. On the basis of these theoretical foundations, we can develop the following exchange rate model, which is specified in Eq. (2):

e=f(i,ms,inf,fxre,bop,rgdp) (2)

From the above theoretical framework, we can develop the model that fulfills one of the objectives of this paper, and is specified in Eq. (3):

rgdp=f(e,i,ms,inf,fxre,bop) (3)

The existence of exchange rate devaluation has both contractionary and expansionary effects on output through the employment effect and the terms of trade balance effects. ^38^ To show which effect will happen when the country devalues its currency; adding nominal exchange rate into the model is necessary. An increase in money supply lowers interests, reduces borrowing costs, and promotes investment which might enhance domestic output. The improvement in investment leads to increase output which intern promotes a country to export more. In addition, a higher money supply will reduce the value of currency. This relation shows money supply affects the balance of payment. ^40^ Thus, we include the money supply in the model. Table 1 summarizes the contractionary effect of devaluation on the basis of various theoretical and empirical studies, highlighting the transmission channels.

**Table 1.** Summary of transmission channels of devaluation.

Number	Channel	Effect of devaluation
1	The balance of payment channel	When a country is initially in a trade deficit and undergoes devaluation, the value of imports surpasses that of exports. The price increase in traded goods reduces the real income of the home country, thereby raising the real income of the rest of the world.
2	Real balance channel	Devaluation results in an increase in the general price level, which leads to a decrease in the real money supply. This reduction in real money supply has the effect of reducing total expenditure and contracting output. In other words, devaluation causes prices to rise, which in turn reduces the purchasing power of money and leads to a decrease in overall spending and economic output.
3	The price channel	Devaluation of a currency results in a higher price of imported goods when measured in the domestic currency. This price increase impacts the demand for domestic products, leading to an increase in demand. Moreover, the price of domestic commodities also tends to rise. In conclusion, devaluation leads to elevated prices of imported goods in the domestic currency, which stimulates the demand for domestic products and creates upward pressure on domestic commodity prices.
5	Interest rate channel	Devaluation can affect the central bank's decision to raise interest rates, leading to an increase in the nominal interest rate and subsequently raising the real interest rate when price expectations and prices remain unchanged in the short term. Conversely, a decrease in the money supply can also raise the real interest rate. This rise in the real interest rate then leads to a decrease in consumption and investment, resulting in a decline in aggregate demand and ultimately decreasing output. In summary, devaluation can cause an increase in the real interest rate, which leads to reduced consumption and investment, ultimately resulting in lower aggregate demand and output.
6	Foreign asset reserve channel	Devaluation leads to an increase in interest rates, which in turn reduces the money supply and causes a decline in stock prices. This scenario hampers firms' ability to finance their activities, as the cost of capital rises due to higher interest rates and increased equity costs associated with issuing stocks. As a result, a decrease in investment is expected, leading to a decline in output. ^44^

**2.2.1 Structural Vector Autoregressive model (SVAR)**

The Structural Vector Autoregressive (here after, SVAR) model is a widely used tool for analysing the monetary transmission mechanism ^45^ and for empirically studying the impact of structural shocks empirically. ^46^^,^^47^ The SVAR model is constructed from the reduced form of the VAR ( *p*) model, which represents the data generated by the structural VAR ( *p*) model, as specified in Eq. (4);

$$B_{0}y_{t}=\alpha_{i,j}+B_{1}A_{i}y_{t‐1}+‐‐‐‐‐‐+B_{P}A_{i}y_{t‐P}$$ (4)

Where, $\alpha_{i,j}$ is an ( n×2 ) matrix constants and linear trends, $B_{i}$ , i=1,…,p, is a K×K matrix of autoregressive slope coefficients, the K×K matrix $B_{0}$ reflects the instantaneous relations among the model variables, and the K×1 vector of mean zero structural shocks $w_{t}$ is serially uncorrelated with a diagonal covariance matrix Σw of full rank such that the number of shocks coincides with the number of variables. The endogenous variable, $\;y_{t}$ , that we include the VAR is the first difference of nominal exchange rate ( $∆e_{t}$ ), output growth ( $∆y_{t}$ ), consumer inflation ( $∆p_{t}$ ), broad money supply ( $∆{\mathrm{ms}}_{t}$ ), nominal interest rate ( $∆i_{t}$ ), foreign exchange reserve ( ∆fxre ), and balance of payment ( bop ). These variables are assumed to be a covariance stationary vector process. For ( $∆e_{t}$ , $∆y_{t}$ , $∆p_{t}$ , $∆{\mathrm{ms}}_{t}\;$ , $∆i_{t}$ , and ∆fxre , we can reject the hypothesis of the existence of a unit root at the 10% level (using Augmented Dickey-Fuller and Phillips-Perron tests). We can, however, not reject the hypothesis of a unit root in bop . Given the low power of these tests in relatively small data sets, we follow ^48^^–^^50^ and assume that bop is stationary since the balance of payment cannot have a unit root at level.

The reduced-form representation of the model in Eq. (5) can be obtained by pre-multiplying both sides of Eq. (4) by $\;{B_{0}}^{-1}$ , results in the model. ^46^^,^^51^^,^^52^

$$y_{t}=\lambda +A_{1}y_{t‐1}+‐‐‐‐‐‐+A_{p}y_{t‐P}+u_{t}$$ (5)

Where; $A_{i}$ = ${B_{0}}^{-1}B_{i}$ and $\;u_{t}={B_{0}}^{-1}w_{t}$ , λ = ${B_{0}}^{-1}\alpha$ and it can be estimated by maximum likelihood (ML) estimation methods.

Estimation of the matrix $B_{0}$ requires additional restrictions on the data generating process (DGP) based on economic theory. If the matrix $B_{0}\;$ can be solved for, given these restrictions and the data, we say that the structural VAR model parameters, $\left(B_{0},B_{1},\ldots ,B_{p},\mathrm{\Sigma w}\right),$ are identified or, equivalently, that the structural shocks $\mathrm{wt}=B_{0}u_{t}$ are identified. In compact form, an SVAR system relates to the following relations as specified in Eq. (6):

$$A_{0}u_{t}={\mathrm{BW}}_{t}$$ (6)

The equation (6) is known as the AB model ^53^; Where $A_{0}\;$ is (n×n) matrix of contemporaneous relations between endogenous variables, *B* is (n×n) matrix that linearly relates the SVAR residuals to the structural innovations, $u_{t}\;$ is vector of reduced-form residual, and $w_{t}\;$ is vector of structural shocks. The residual $u_{t}\;$ in the reduced form is presumed to be white noise. Therefore, we can estimate the AB model by OLS (ordinary list square).

The structural innovations $w_{t}$ can be derived from errors $u_{t}$ of the reduced form, but certain restrictions must be placed on the system. In details, $\;\frac{n\left(n-1\right)}{2}\;$ [ 1] Where n is the number of variables in the model; restrictions must be imposed on *A*0 matrix to be able to identify the structural shocks. ^54^

**2.2.2 Recursively identified structural VAR model**

In this study, based on the work of, ^46^ the response of economic variables to temporary shocks was estimated using a recursively identified structural model. This approach assumes that the current structural shocks are not influenced by the preceding ordering variables. Instead, it is assumed that the variables are affected by a sequential chain of shocks, or alternatively, the matrix $A_{0}$ is diagonal, indicating that the structural shocks are orthogonal. Specifically, the matrix $A_{0}$ takes the form of a lower triangular matrix, as illustrated below:

$$\left(\begin{array}{c} {u_{t}}^{e}\\ {u_{t}}^{i}\\ \begin{array}{c} {u_{t}}^{\mathrm{ms}}\\ {u_{t}}^{\inf}\\ \begin{array}{c} {u_{t}}^{\text{fxre}}\\ {u_{t}}^{\mathrm{bop}}\\ {u_{t}}^{\text{rgdp}} \end{array} \end{array} \end{array}\right)=\left[\begin{array}{ccccccc} 1 & 0 & 0 & 0 & 0 & 0 & 0\\ b_{21} & 1 & 0 & 0 & 0 & 0 & 0\\ b_{31} & b_{32} & 1 & 0 & 0 & 0 & 0\\ b_{41} & b_{42} & b_{43} & 1 & 0 & 0 & 0\\ b_{51} & b_{52} & b_{53} & b_{54} & 1 & 0 & 0\\ b_{61} & b_{62} & b_{63} & b_{64} & b_{65} & 1 & 0\\ b_{71} & b_{72} & b_{73} & b_{74} & b_{75} & b_{76} & 1 \end{array}\;\right]*\left(\begin{array}{c} {w_{t}}^{e}\\ {w_{t}}^{i}\\ \begin{array}{c} {w_{t}}^{\mathrm{ms}}\\ {w_{t}}^{\inf}\\ \begin{array}{c} {w_{t}}^{\text{fxre}}\\ {w_{t}}^{\mathrm{bop}}\\ {w_{t}}^{\text{rgdp}} \end{array} \end{array} \end{array}\right)$$

Where, $\left({u_{t}}^{e},{u_{t}}^{i},{u_{t}}^{\mathrm{ms}},{u_{t}}^{\inf},{u_{t}}^{\text{fxre}},{u_{t}}^{\mathrm{bop}}\;\text{and}\;{u_{t}}^{\text{rgdp}}\right)$ , are the structural disturbance, that is, exchange rate shocks, interest rate shocks, money supply, the price shocks, foreign asset reserve shocks, the balance of payment shocks, and output shocks respectively; and ( ${w_{t}}^{e},{w_{t}}^{i},{w_{t}}^{\mathrm{ms}},{w_{t}}^{\inf},{w_{t}}^{\text{fxre}},{w_{t}}^{\mathrm{bop}},\text{and}\;{w_{t}}^{\text{rgdp}})$ , are the residuals in the reduced form equations, which represent unexpected movements (given information in the system) of each variable.

In certain scenarios where a fully developed theoretical model is unavailable, the process of identification can be achieved through the incorporation of extraneous information and the selective utilization of insights from economic theory. In line with, ^46^ we present the construction of a recursive form of the SVAR (Structural Vector Autoregressive) model, as demonstrated above.

Once the structural shocks have been identified, a rigorous analysis and interpretation of these macroeconomic shocks become imperative. This analysis is conducted within the framework of structural VAR models and involves the examination of structural impulse responses as well as the implementation of forecast error variance decompositions. These analytical techniques provide valuable insights into the dynamics and impacts of the identified shocks.

## 3. Results and Discussion

### 3.1 Stationarity test

To enhance the robustness of the test, the presence of a unit root was assessed using the Augmented Dickey–Fuller (ADF) and Phillip-Perron (PP) tests. The results of the unit root tests conducted using the Augmented Dickey–Fuller and Phillip-Perron methods are summarized in Table 2. Based on the results of the ADF and PP tests, it is evident that all variables in the VAR model, except bop, are nonstationary at this level. This implies that the null hypothesis of a unit root, including both trend, and trend and intercept, cannot be rejected for these variables. However, upon considering their first differences, these variables exhibit stationarity.

**Table 2.** Augmented Dickey Fuller and Phillip-Perron (PP) unit root test.

	ADF test statistics		PP test statistics	
Variables	At level	At first difference	At level	At first difference
*e*	0.375583	-5.551334***	4.519163	-5.610420***
*i*	-2.324670	-9.304296***	-2.324670	-9.303151***
*lnms*	-3.976900	-9.777815***	-3.921847	-12.41923***
*inf*	-2.438531	-4.431002***	-3.320427	-5.910959***
*lnfxre*	-1.226036	-9.962558***	-1.226036	-9.960837***
*bop*	-6.294778***	-12.43021***	-6.303920***	-26.733448***
*lnrgdp*	-2.338523	-9.270263***	-2.386307	-9270263***

Source: Author’s estimation.

Note: *, **, *** represents significant at 10%, 5% and 1% level of significance respectively.

### 3.2 Lag Length and stability check

o determine the lag length of VAR/SVAR, the study employs different lag-length selection criteria, including the likelihood ratio test statistic (LR), Final Prediction Error (FPE), Akaike Information Criteria (AIC), and the Hannan-Quinn Information Criterion (HQ). In Table 3 the lag length selection criterion is tabulated. The AIC lag length section test indicates that the appropriate lag length for the VAR model is three (3).

**Table 3.** VAR lag order selection criteria.

Sample: 2001q1 thru 2023q4						Number of obs = 88		
Lag	LL	LR	df	p	FPE	AIC	HQIC	SBIC
0	-1163.84		49	0.000	849.751	26.6101	26.6895	26.8071
1	-398.009	1531.7	49	0.000	.000072	10.3184	10.9535 *	11.8949 *
2	-333.085	129.85	49	0.000	.000051	9.95648	11.1473	12.9124
3	-280.034	106.1	49	0.000	.000049 *	9.86442 *	11.611	14.1998
4	-233.361	93.346 *	49	0.000	.000057	9.91731	12.2196	15.6321

Source: Author’s estimation.

* Optimal lag length selected by the criterion.

Prior to estimating the parameters of the SVAR model with the optimal lag length, it is essential to assess the stability conditions of the VAR model by examining the AR roots. The obtained results, which are reported in Table 4, indicate that all eigenvalues in the proposed model lie within the unit circle, signifying values that are either less than one or equal to unity. Consequently, it can be concluded that the VAR/SVAR model satisfies the requisite stability condition.

**Table 4.** Roots of characteristic polynomial (Eigenvalue stability condition).

.9965398 + .02269096i	.996798
.9965398 - .02269096i	.996798
.9340958 + .1690577i	.949271
.9340958 - .1690577i	.949271
.7510523 + .3282537i	.819652
.7510523 - .3282537i	.819652
.7062889	.706289
-.1773563 + .6833501i	.705991
-.1773563 - .6833501i	.705991
.6681919 + .2093041i	.700206
.6681919 - .2093041i	.700206
.231783 + .6051372i	.648008
.231783 - .6051372i	.648008
.5513563	.551356
-.5053132	.505313
-.4430517 + .2106936i	.490598
-.4430517 - .2106936i	.490598
-.2550144	.255014
-.04897661 + .2470312i	.251839
-.04897661 - .2470312i	.251839
-.1851126	.185113

All the eigenvalues lie inside the unit circle.

VAR satisfies stability condition.

Source: Author’s estimation.

### 3.3 Co-integration test and post estimation test

The unit root test reveals that the model in our study consists of a mixture of I(1) and I(0) variables. To investigate whether these variables are cointegrated at the same order (only I(1) variables) and at different orders (I(0) and I(1)), we employ Johansen’s cointegration technique. ^36^ Our analysis is based on an SVAR model with a mixture of I(1) and I(0) variables, as described previously. ^48^^,^^55^^,^^56^ The results of Johansen’s cointegration test in Table 5 indicate the presence of multiple cointegrating equations and vectors in the case of I(1) variables, whereas for the mixture of I(0) and I(1), evidence suggests the presence of three cointegrating relationships. These findings contribute to a deeper understanding of the long-run dynamics and interdependencies among the macroeconomic variables studied.

**Table 5.** Johansen tests for cointegration.

Trend: Constant Sample: 2001q1 thru 2023q4				Number of obs = 89 Number of lags = 3	
Maximum rank	Params	LL	Eigenvalue	Trace statistic	Critical value
0	105	-357.14473	.	148.4717	124.24
1	118	-332.78126	0.42160	99.7447	94.15
2	129	-311.68026	0.37760	57.5428 *	68.52
3	138	-298.95665	0.24868	32.0955	47.21
4	145	-292.025	0.14424	18.2322	29.68
5	150	-286.2986	0.12075	6.7794	15.41
6	153	-283.74009	0.05587	1.6624	3.76
7	154	-282.90888	0.01851		

* Selected rank.

The study investigated the presence of serial autocorrelation ( Table 6) before discussing the impulse response function. The selected lags in the VAR model exhibit no significant serial autocorrelation at the 5% level, and the parameter estimates remain stable. As a result, the SVAR models with three lags satisfy the stability condition and can be reliably estimated.

**Table 6.** Serial autocorrelation test (Lagrange-multiplier test).

lag	chi ^2^	df	Prob > Chi ^2^
1	57.449	49	0.191
2	46.266	49	0.585

Ho: no autocorrelation at lag order.

Source: Own computation.

### 3.5 Impulse response and variance decomposition

**3.5.1 The response of the balance of payment to shocks**

**Figure 1.**
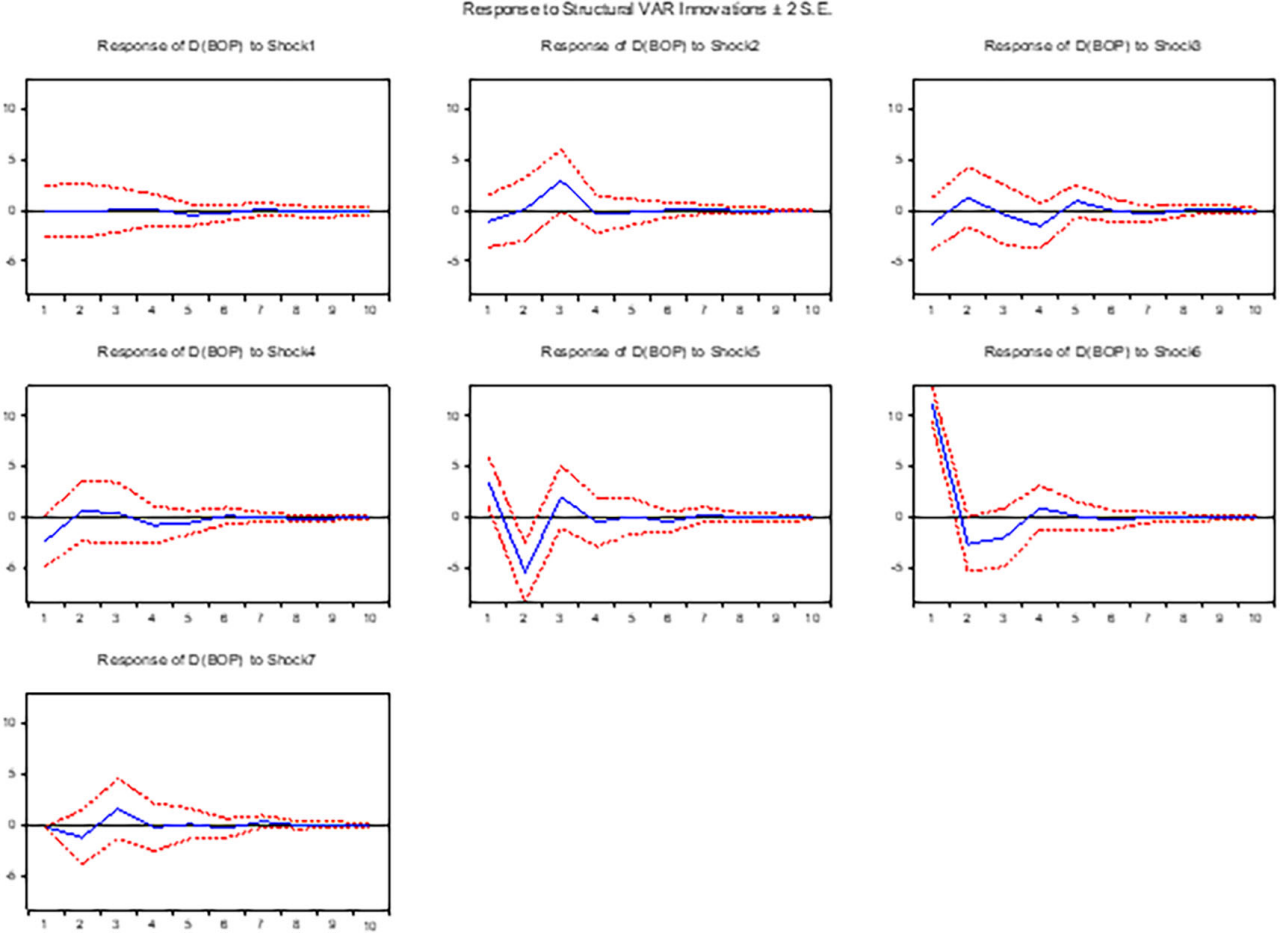
The response of the balance of payment to exchange rate and other macroeconomic shocks in the long run.

Figure 1 illustrates positive shocks on the exchange rate and other variables included in the balance of payment channel in the long run. In both short run and long run, devaluation negatively affects the balance of payment. Contrary to initial expectations, our findings indicate that devaluation does not have a significant long-run impact. Therefore, we reject the null hypothesis suggesting that devaluation enhances the balance of payments in the long run. Interestingly, we discover that a positive shock exchange rate actually has a negative effect on the balance of payments. The success of devaluation as a strategy to improve the balance of payments depends on the elasticities of exports and imports. In the context of Ethiopia, where elasticity is significantly low, devaluation is not expected to lead to an improvement in the balance of payments. These findings align with those of previous studies conducted by. ^57^^,^^58^

Additionally, we find that an initial positive shock in interest rates improves the balance of payments, but this effect diminishes after the fourth quarter in both the short and long run. On the other hand, in the short run, a positive innovation in money supply negatively affects the balance of payments up to the fourth quarter, and after the fourth quarter, its effect is positive. However, in the long run, a positive shock in the money supply initially enhances the balance of payments, but the effect diminishes after the fourth quarter in the long run. Furthermore, the response of the balance of payments to a positive innovation in inflation is negative in the short run and positive up to the third quarter. However, after the fourth quarter, the balance of payments worsens in the long run, which contradicts the findings of. ^59^ This divergence highlights the need for further investigation into the dynamics of inflation and its impact on the balance of payments in Ethiopia. Lastly, a positive shock in foreign asset reserves affects negatively up to the third quarter and positively after the third quarter in both the short and long run. Initially, a positive shock on real output has a negative impact on the balance of payments both in the short and long run up to the second quarter; thereafter, its impact is positive.

**3.5.2 The response of real GDP (output growth) to shocks**

**Figure 2.**
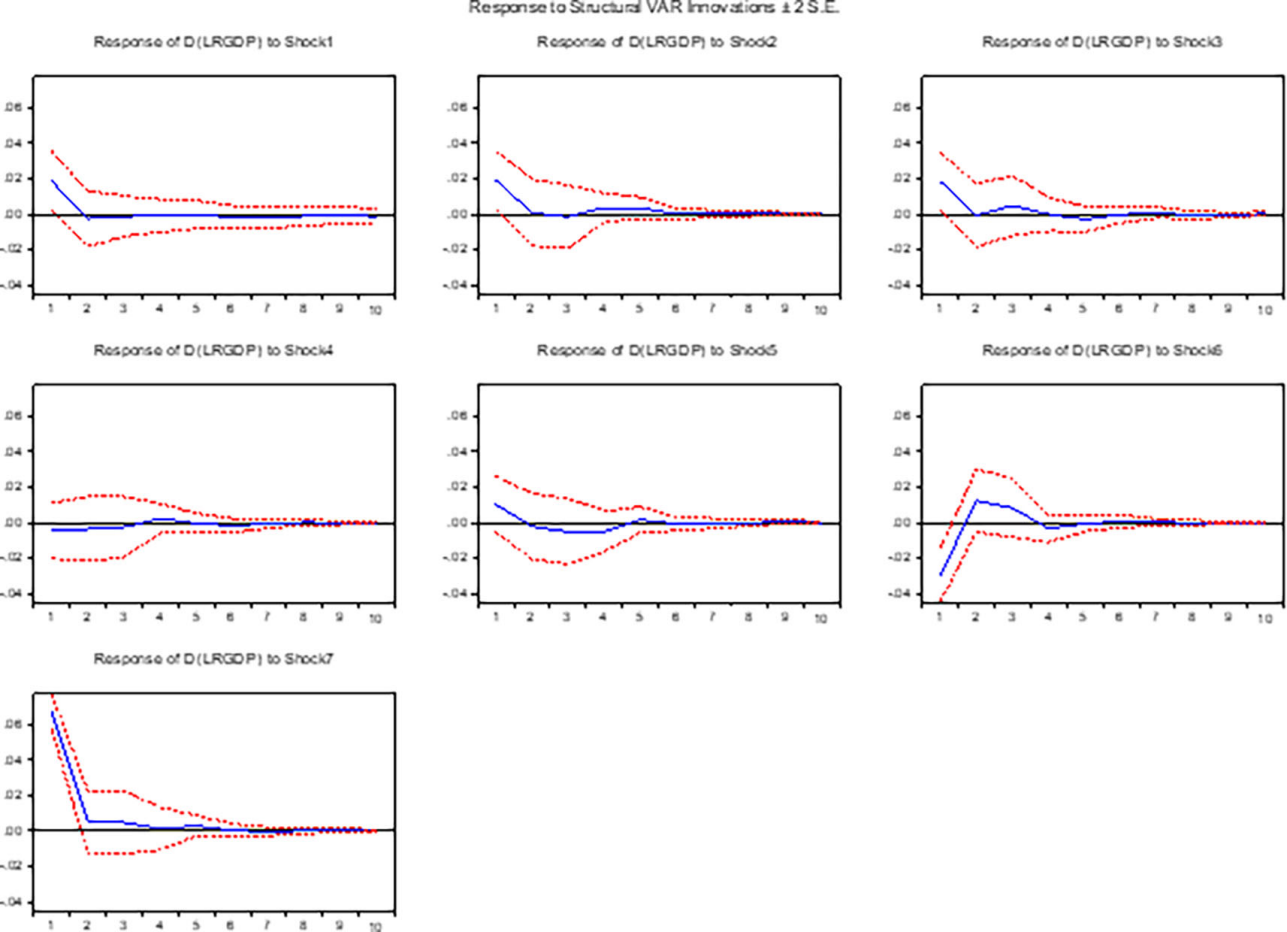
The response of output growth to exchange rate and other macroeconomic shocks in the long run.

Figure 2 presents empirical evidence on the contemporaneous response of output to various macroeconomic shocks, including positive innovations in the nominal exchange rate, interest rate, money supply, inflation, foreign asset reserves, and balance of payments in the long run. Our analysis reveals that the nominal exchange rate has a negative impact on output in both short run and long run, indicating a contractionary effect. These findings are consistent with prior research by, ^15^^,^^32^^,^^60^ providing further support for the hypothesis that positive shocks to the exchange rate adversely affect output. Additionally, we find empirical evidence supporting the null hypothesis, confirming that devaluation has a contractionary effect on output.

Furthermore, our study reveals that positive innovations in interest rates have a negative effect on output both in the short and long run, providing empirical support for the monetary theory. This finding strengthens the existing literature by confirming the negative relationship between output and interest rates. Moreover, our analysis reveals that a positive shock in the money supply leads to a positive response in output. This observation suggests that an increase in money supply stimulates output, thereby contributing to economic growth in both the short and long run.

In line with the research conducted by ^59^ our study highlights the adverse impact of inflation on output against the findings of. ^12^ Specifically, we find that an increase in inflation significantly and negatively affects output in both the short and long run, emphasizing the detrimental consequences of rising inflation for overall economic performance. Regarding foreign asset reserves, our analysis indicates that an initial positive shock in these reserves adversely affects output in both the short and long run. However, after three quarters in the long run, the effect becomes positive, suggesting a gradual recovery and the potential for long-term benefits. This dynamic nature of the relationship between foreign asset reserves and output in the long run underscores the importance of considering the timing and persistence of shocks when assessing their effects. In addition, our study reveals a positive contemporaneous response of output to positive shocks in the balance of payments. Specifically, a surplus in the balance of payments improves the level of output, indicating a favourable impact on the economy.

In summary, the findings confirm the contractionary effect of positive shocks in the nominal exchange rate, the inverse relationship between output and interest rates, and the positive impact of money supply shocks on output. Furthermore, our study emphasizes the negative effects of inflation on output and highlights the dynamic nature of the relationship between foreign asset reserves and output.

**3.5.3 The response of money supply to shocks**

**Figure 3.**
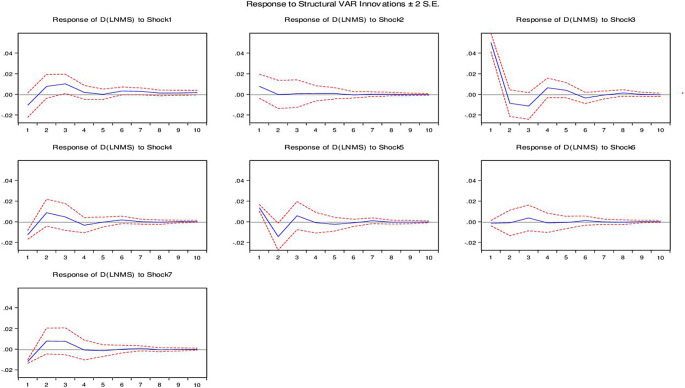
The response of money supply (M2) to exchange rate and other macroeconomic shocks in the long run.

Figure 3 illustrates the response of the money supply (M2) to contemporaneous positive shocks in macroeconomic variables. Specifically, we examine the response of the money supply to positive innovations in the nominal exchange rate, interest rate, inflation, foreign exchange reserves, balance of payments, and output. In the case of a positive innovation in the nominal exchange rate, our findings reveal that the response of the money supply is positive during the first three quarters in both the short and long run. This implies that an initial positive shock to the nominal exchange rate leads to an increase in the money supply in Ethiopia. However, after the third quarter, the response of the money supply declined, although it remained positive. This suggests a diminishing effect over time. Consistent with economic theory, our analysis supports the inverse relationship between the interest rate and money supply in Ethiopia. A positive innovation in the interest rate leads to a decrease in the money supply, aligning with the expected relationship.

Furthermore, our findings indicate that a positive innovation in inflation has a significant effect on increasing the money supply in Ethiopia. This suggests a positive relationship between inflation and money supply, highlighting the impact of inflation on the monetary dynamics of the Ethiopian economy. Regarding foreign exchange reserves, our analysis reveals a dynamic response of money supply. A positive shock in foreign exchange reserves initially leads to an increase in money supply. However, similar to the response to the nominal exchange rate, the effect gradually declines over time. Moreover, our study finds that a positive shock in the balance of payments leads to an increase in money supply in Ethiopia. This implies a positive relationship between the balance of payments and money supply, indicating the influence of external factors on the domestic money supply dynamics in the Ethiopian context in both short run and long run.

Finally, our analysis demonstrates a positive response of money supply to positive shocks in output. This implicates that a positive shock to output leads to a rise in money supply in Ethiopia, highlighting the relationship between output and money supply dynamics in the country.

**3.5.4 The response of inflation**

**Figure 4.**
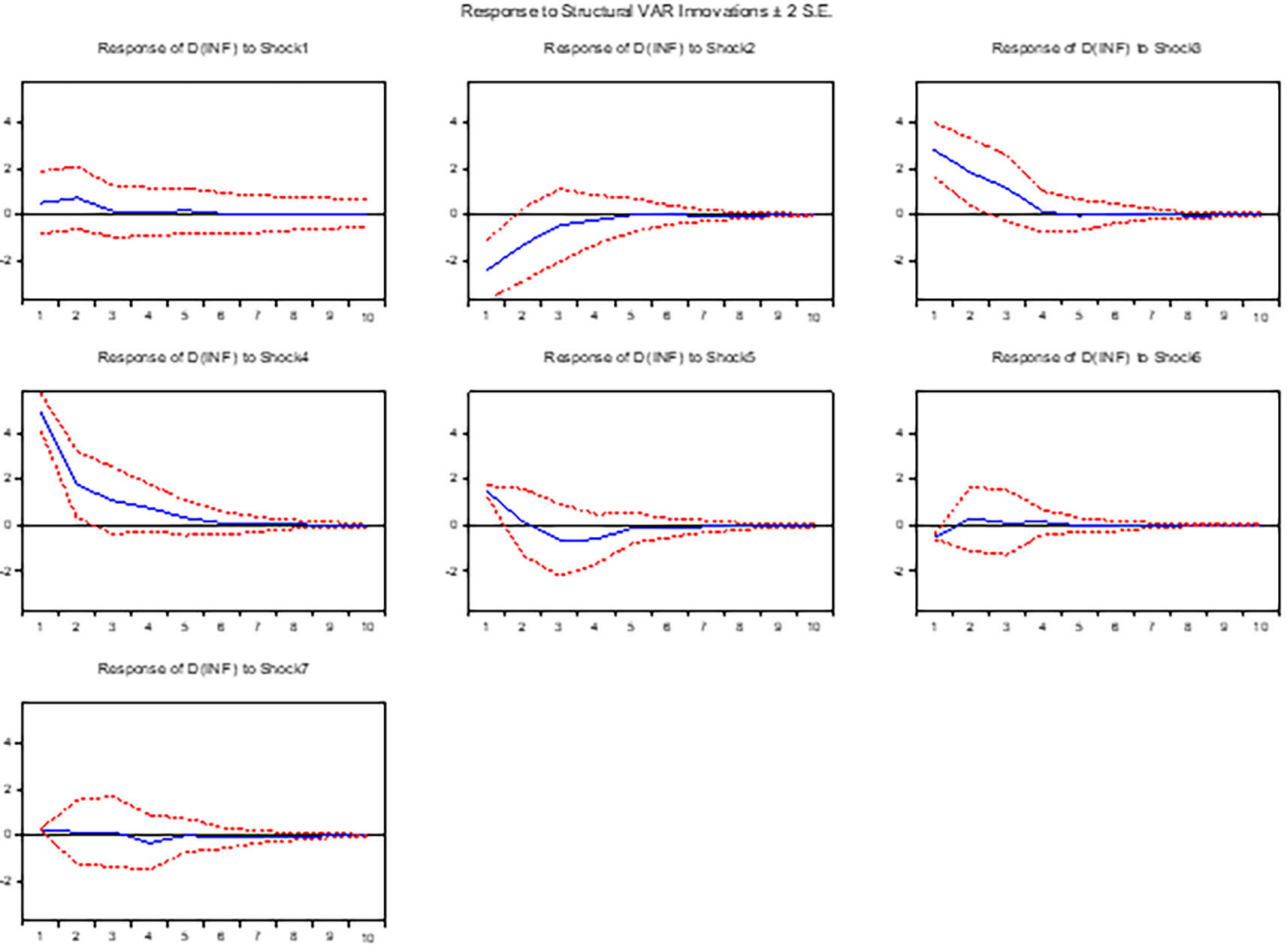
The response of inflation to exchange rate and other macroeconomic shocks in the long run.

Figure 4 presents the impulse response analysis of inflation to exogenous shocks in the innovations of other variables within the system. Specifically, we examine the effects of a positive shock in the exchange rate, interest rate, and money supply on inflation dynamics. Our findings reveal that a positive shock in the exchange rate, indicative of devaluation in the domestic currency, exerts a positive impact on inflation in both the short and long run. This outcome suggests the presence of inflationary pressures resulting from exchange rate movements, whereby devaluation in the domestic currency contributes to higher import costs and subsequently increases domestic prices. ^6^ In contrast, the response of inflation to a positive random shock in the innovation of the interest rate demonstrates a negative relationship. This observation implies that an increase in interest rates reduces inflationary pressures within the economy. Moreover, a positive random shock in the money supply is associated with an increase in inflation. This result suggests that an expansionary monetary policy, as reflected by an increase in the money supply, contributes to higher inflationary pressures.

Overall, these findings underscore the interconnectedness and dynamics of inflation with respect to exogenous shocks in the exchange rate, interest rate, and money supply. The implications of these relationships provide valuable insights for policymakers in managing inflationary pressures and devising appropriate monetary and exchange rate policies.

**3.5.5 The response of foreign exchange reserve**

**Figure 5.**
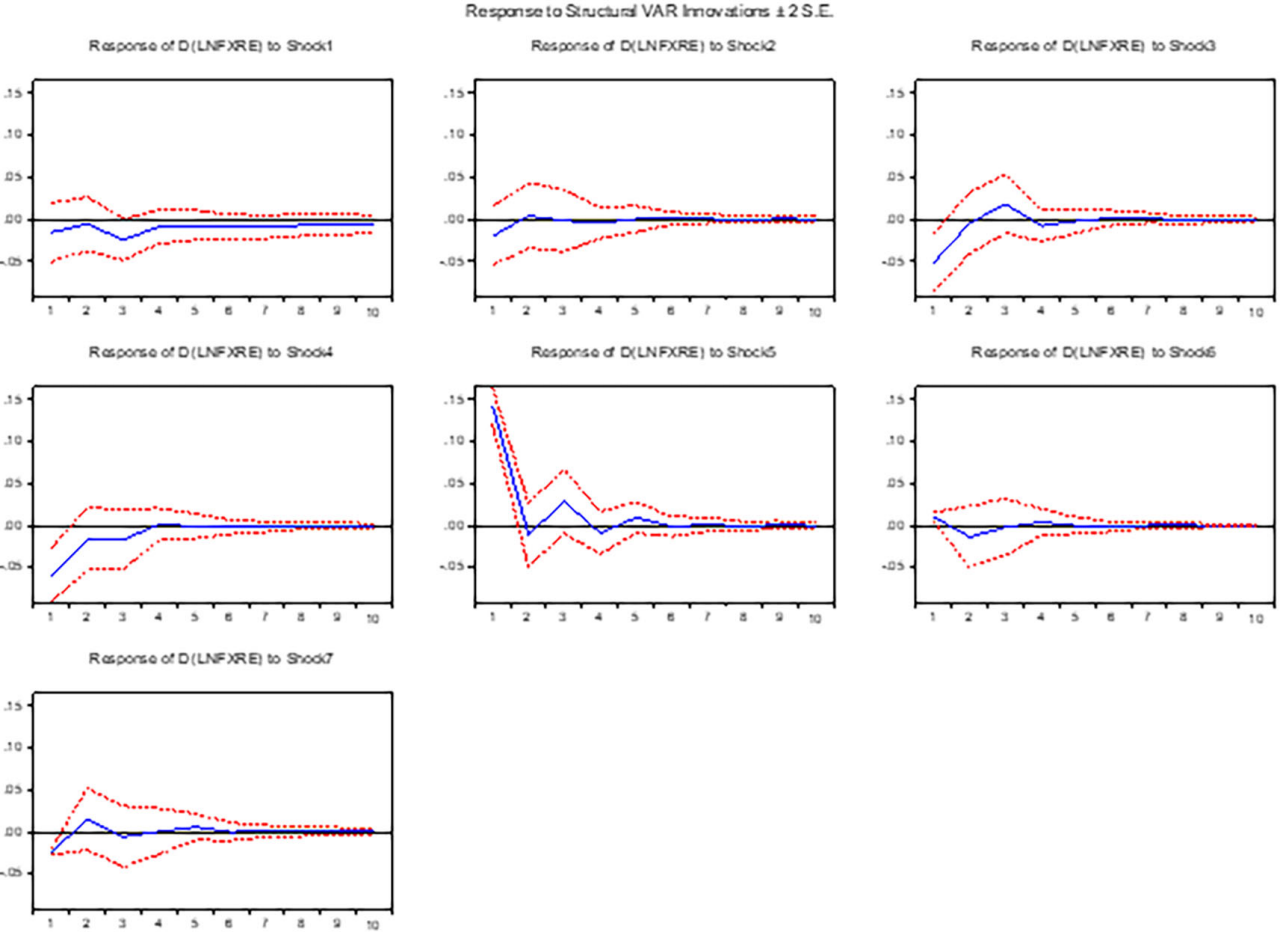
The response of foreign asset reserve to exchange rate and other macroeconomic shocks in the long run.

Figure 5 presents the impact of devaluation and other macroeconomic variables on foreign asset reserves. Devaluation of the domestic currency leads to a decline in foreign asset reserves. The response of reserves to positive innovations in the interest rate initially exhibits a negative relationship, but after a few quarters of adjustment, it results in an increase in reserves. Moreover, the response of foreign asset reserves to changes in the money supply is positive, indicating that an expansionary monetary policy contributes to an increase in reserves.

This study also highlights the positive relationship between inflationary shocks and foreign asset reserves. When inflationary pressures arise, the central authority takes measures to collect funds to mitigate inflation. Consequently, the reserve in banks increases. Furthermore, lagged positive innovations have a significant impact on the current performance of foreign asset reserves. These findings shed light on the dynamics and interplay among currency devaluation, interest rate innovations, money supply changes, inflationary shocks, and foreign asset reserves. The implications of these relationships provide valuable insights for policymakers in managing and optimizing foreign asset reserves in response to various economic shocks and policy interventions.

**3.5.6 The response of interest rate**

**Figure 6.**
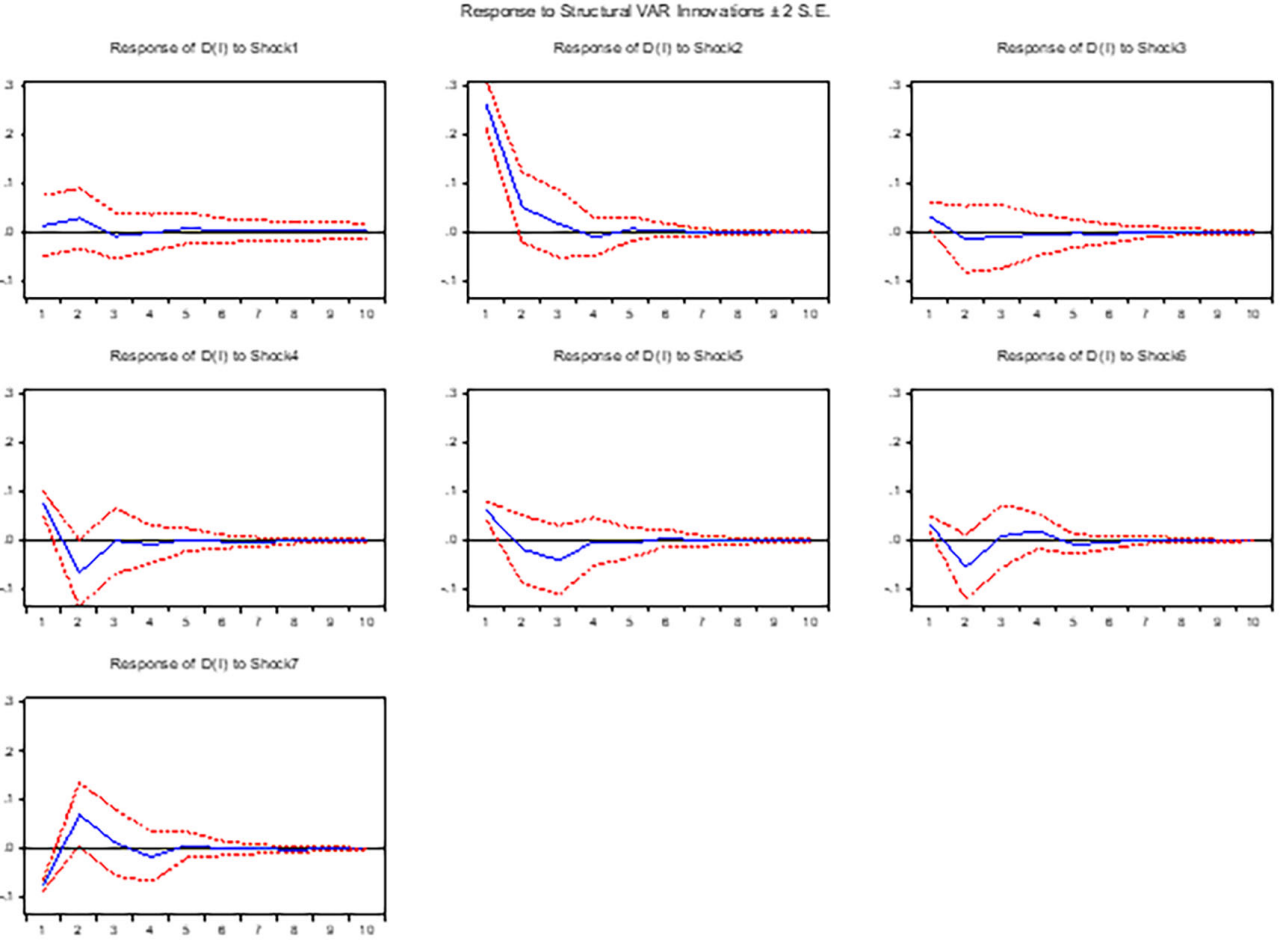
The response of interest rate to exchange rate and other macroeconomic shocks in the long run.

Figure 6 shows the responses of the interest rate to positive shocks in the innovations of other variables in the system. Initially, positive shocks on the exchange rate have a positive effect, but the effect declines after the second quarter. The response of the interest rate to the money supply is negative. An increase in inflation leads to a decline in the interest rate, and the effects of foreign asset reserves initially increase the interest rate, but the interest rate declines a few quarters later. The balance of payment surplus influenced the domestic interest rate to decline. Responses to positive innovation in output led to an increase in the lending interest rate.

**3.5.7 Estimation results of Variance decomposition**

The variance decomposition analysis of the balance of payment (bop) and output (lrgdp) is used to examine the strengths of various devaluation channels. The study includes forecast horizons ranging from 1 to 10 quarters. The first column of the results displays the forecast periods, while the second column represents the standard error (SE), indicating the forecast error at different quarters. Shocks denoted as Shock1 (e), Shock2 (i), Shock3 (lnms), Shock4 (inf), Shock5 (lnfxre), Shock6 (bop), and Shock7 (lrgdp) are used to represent each variable. Furthermore, the decomposition values for the 1st to 10th forecast horizons are presented in Tables 7 and 8.

**Table 7.** Estimation results of Variance decomposition of balance of payment.

Variance Decomposition of D (BOP):								
Period	S.E.	Shock1	Shock2	Shock3	Shock4	Shock5	Shock6	Shock7
1	12.10029	0.007171	0.775792	1.183007	3.427405	8.171168	86.40430	0.031155
2	13.62480	0.006623	0.619639	1.852752	2.926078	21.95250	71.77677	0.865642
3	14.32434	0.006715	4.805973	1.769181	2.754306	21.83014	66.79856	2.035130
4	14.47296	0.010017	4.782276	2.880890	2.941963	21.47061	65.89581	2.018434
5	14.51992	0.101340	4.764648	3.270370	3.031424	21.33942	65.48312	2.009677
6	14.53482	0.127391	4.755807	3.263699	3.041905	21.37663	65.36825	2.066318
7	14.54815	0.132023	4.754523	3.321339	3.037411	21.39368	65.24867	2.112349
8	14.54890	0.135319	4.754546	3.321143	3.042992	21.39170	65.24199	2.112315
9	14.55104	0.147741	4.753294	3.334787	3.043872	21.38545	65.22292	2.111940
10	14.55154	0.149596	4.753062	3.337253	3.044390	21.38521	65.21857	2.111921

Source: Own computation.

**Table 8.** Estimation results of Variance decomposition of balance of payment.

Variance Decomposition of D (LRGDP):								
Period	S.E.	Shock1	Shock2	Shock3	Shock4	Shock5	Shock6	Shock7
1	0.08087	5.239407	5.505046	5.197585	0.337055	1.586882	13.34991	68.78411
2	0.08210	5.175452	5.351709	5.048614	0.513015	1.609705	15.20399	67.09752
3	0.08302	5.078412	5.261189	5.274374	0.591353	1.968570	15.87024	65.95587
4	0.08336	5.049644	5.411485	5.231688	0.674714	2.283209	15.89755	65.45171
5	0.08355	5.027476	5.554893	5.323698	0.672986	2.313332	15.83107	65.27654
6	0.08359	5.063252	5.549766	5.318604	0.714668	2.317426	15.82381	65.21247
7	0.08363	5.115420	5.545683	5.327873	0.718533	2.319310	15.81137	65.16181
8	0.08364	5.129035	5.544870	5.334826	0.718857	2.318975	15.80750	65.14593
9	0.08365	5.137366	5.544407	5.336252	0.719256	2.321676	15.80449	65.13656
10	0.08365	5.153004	5.543404	5.336124	0.720600	2.321255	15.80149	65.12412

Source: Own computation.

The results of our variance decomposition analysis in the short-run shocks on the exchange rate significantly affect the balance of payment next to the foreign exchange reserve. In the long run, foreign exchange rate reserve shocks (Shock 5) have more significant explanatory power in accounting for balance of payment (bop) fluctuations than nominal exchange rate shocks (Shock 1) and output shocks (Shock 7). Thus, the foreign exchange rate reserve channel emerges as a dominant factor in the context of exchange rate policy and its influence on balance of payment dynamics. In contrast, the impact of the exchange rate channel alone appears to be ineffective in the long run, as indicated by its contribution to the balance of payment fluctuations in Table 7. However, the exchange rate channel indirectly affects the balance of payment through other channels, such as the foreign exchange reserve, output, and money supply channels. This highlights the interdependencies and interconnectedness of these channels in shaping balance of payment dynamics in the long run.

The results of our variance decomposition analysis in Table 8 indicate that balance of payment shocks (Shock 6), interest rate shocks (Shock 2), exchange rate shocks (Shock 1), and money supply shocks (Shock 3) have a significant impact on output variations in the long run. Among these shocks, the exchange rate channel emerges as a crucial factor that interacts with the balance of payment and interest rate channels, shaping the dynamics of output fluctuations.

## 4. Conclusion and policy recommendations

This study addresses the devaluation puzzle by providing a more comprehensive and nuanced understanding of how devaluation affects the balance of payments and output. To achieve this, we employ a recursive structural vector autoregressive (SVAR) model focusing on Ethiopia from 2001Q1 to 2023Q4. In addition to examining the exchange rate, we incorporate other essential variables such as foreign asset reserves, interest rates, money supply, and inflation. By including these variables, we provide a more complete depiction of the transmission channel. This study also demonstrated unit root tests using the ADF and PP tests, model stability, and other necessary assessments.

The findings of this study lead to the following key conclusions: first, a shock to the exchange rate hurts the balance of payments in both the short and long run. The effectiveness of exchange rate movements on the balance of payments is greater in the short run than eventually. Second, shocks to other variables such as foreign exchange rate reserves, inflation, and interest rates exert a stronger influence on the balance of payments than the exchange rate itself in the long run. Moreover, in the long run, shocks in the exchange rate have a significant impact on foreign exchange rate reserves. The impact of devaluation on the balance of payments, particularly through the exchange rate channel, is limited when considered in isolation over the long run. Instead, its influence is observed indirectly through interactions with other channels, such as the foreign exchange reserve channel, highlighting the complex dynamics of exchange rates. Third, shocks to the exchange rate hurt output growth, both in the short and long run. Fourth, in the long run, the exchange rate responds negatively to shocks in the balance of payments and output growth (appreciation of domestic currency). Changes in the balance of payments and output explain a significant portion of the variability in exchange rate movements. Finally, the study concludes that devaluation has a contractionary effect through various channels. Devaluation increases the money supply, leading to inflationary pressures and a decline in output. It also increases interest rates, which further reduces output. Additionally, devaluation reduces foreign asset reserves, resulting in a fall in the balance of payments and a decrease in output.

Based on the findings of this study, several policy recommendations arise: first, given the negative impact of exchange rate shocks on the balance of payments and output growth, policymakers should prioritize maintaining exchange rate stability. This can be achieved through prudent monetary and fiscal policies that avoid excessive exchange rate volatility and promote economic stability. Second, because shocks in the exchange rate significantly affect foreign exchange rate reserves, the National Bank of Ethiopia should diversify its reserve holdings. Holding a diversified portfolio of currencies and assets can help mitigate the adverse effects of exchange rate fluctuations and enhance the resilience of the balance of payments. Third, while the exchange rate plays a role in the balance of payments, policymakers should adopt a broader perspective by considering the interplay of other variables such as foreign exchange rate reserves, inflation, and interest rates. Implementing comprehensive economic policies that address these factors can significantly impact the balance of payments in the long run. Fourth, policymakers should prioritize effective inflation control.

This involves implementing appropriate monetary policies to control money supply growth, ensuring price stability, and minimizing the inflationary pressures associated with devaluation. Fifth, given the negative impact of exchange rate shocks on the balance of payments, policymakers should focus on enhancing the competitiveness of domestic industries in global markets. This can be achieved by investing in infrastructure, providing targeted support to export-oriented sectors, and implementing policies that foster innovation and productivity improvements.

Finally, it is recommended that international financial institutions such as the International Monetary Fund (IMF) and the World Bank revisit their policy advising developing countries to devalue their currencies. In addition, the National Bank of Ethiopia should reassess its policies that prioritize currency devaluation as the preferred approach for the development of the economy. These revisions should consider the complexities and potential negative consequences associated with devaluation.

## Data Availability

Zenodo: Unravelling the Devaluation Puzzle: Empirical Insights into the Transmission Channel on Balance of Payments and Output in Ethiopia https://zenodo.org/doi/10.5281/zenodo.11190281. This project contains the following underlying data:
-
data.xlsx

## Footnotes

^1^
The contemporaneous matrix requires $\left(\frac{7\left(7-1\right)}{2}=24\right)$ restrictions for exact identification more than 24 restrictions were imposed, leading to over identification *.*

## Software availability

Available from: https://www.google.com/url?sa=t&source=web&rct=j&opi=89978449&url=https://www.stata.com/stata17/&ved=2ahUKEwi03vfG_pGGAxUzgv0HHQ7yC2sQFnoECC4QAQ&usg=AOvVaw03lYw5vn_Q1Y0pZIr3mJKg

License: ©Copyright 1996–2024 StataCorp LLC

https://www.google.com/url?sa=t&source=web&rct=j&opi=89978449&url=https://www.eviews.com/download/ student12/&ved=2ahUKEwjfiguqwJnl_pGGAxVLg_0HHToUAGIQjBB6BAgaEAE&usg=AOvVaw30-y1H1GZcVbJaDDX-SPiz

License: ©Copyright 2022 EViews, IHS Global Inc.
