# Unravelling the devaluation puzzle: Empirical insights into the transmission channel on balance of payments and output in Ethiopia

**DOI:** 10.12688/f1000research.151984.2

**Published:** 2025-05-07

**Authors:** Yigermal Maru Ayinewa, Mesele Belay Zegeye, Tesfahun Ayanaw Alemu, Abate Belaye Tefera

**Affiliations:** 1 Economics, Woldia University, Weldiya, Amhara, Ethiopia

**Keywords:** Devaluation, Balance of Payments, Output growth, SVAR Model, Transmission Channels

## Abstract

**Background:**

Empirical studies on the impact of devaluation in developing countries, including Ethiopia, have revealed diverse and mixed results. The effects can be positive or negative depending on the specific economic context and policies in place.This study addresses the devaluation puzzle by providing a more comprehensive and nuanced understanding of how devaluation affects the balance of payments and output.

**Methods:**

To achieve this, we employ a recursive structural vector autoregressive (SVAR) model focusing on Ethiopia from 2001Q1 to 2023Q4.

**Results:**

The estimation results indicate that the real effective exchange rate channel effectively influences both the balance of payments and output in Ethiopia. Additionally, while foreign asset reserves, money supply, and inflation channels have a stronger impact on output, their effect on the balance of payments is relatively weak. The analysis further indicates that currency depreciation can improve the BOP in the short period by enhancing export competitiveness; however, it may have negative long-period effects due to rising import prices. Similarly, although an increased money supply can stimulate economic activity and strengthen the BOP, excessive expansion risks inflation and trade deficits. Furthermore, inflation negatively impacts both the BOP and GDP by escalating import costs and diminishing competitiveness. Foreign exchange reserves play a crucial role in supporting external liquidity and investor confidence, essential for economic stability.

**Conclusion:**

The interconnectedness of these factors emphasizes the need for policymakers to implement effective management strategies, including enhancing foreign reserve management, controlling inflation, and adopting a balanced exchange rate policy. Continuous monitoring of these policies will help address emerging challenges and improve Ethiopia’s competitiveness in the global market.

## 1. Introduction

This study examines the complex dynamics between devaluation, the balance of payments, and output in Ethiopia. Devaluation, a decline in the value of a country’s currency relative to foreign currency, is recognized as a tool for stabilizing the foreign sector of an economy. ^1^^–^^3^ During the 1970s and 1980s, many developing countries faced severe balance of payments deterioration due to overvalued currencies and high unemployment rates. ^4^ Consequently, these countries sought assistance from international organizations, such as the International Monetary Fund (IMF) and the World Bank (WB), both of which were cautious in providing aid without specific conditions, including the implementation of structural adjustment programs (SAPs). ^5^^,^^6^ As a result, numerous African countries, including Ethiopia, agreed to implement SAPs and fulfil the World Bank and IMF requirements.

The impact of exchange rate movements in developing countries varies depending on the nature of their principal exports. It is governed by the Marshall-Lerner condition, which determines the effect of exchange rate depreciation or appreciation on the balance of payment. ^7^

Since 1992, Ethiopia has followed a managed floating exchange rate system, which has resulted in significant fluctuations in the official exchange rate. One notable instance of devaluation occurred when the exchange rate surged from 2.07 birr/dollar to 5 birr/dollar, representing a remarkable devaluation rate of 142%, the highest in Ethiopian history. In September 2010, there was another significant increase in the exchange rate, with a rise of 16.7% from 13.6 birr/dollar to 16.3 birr/dollar. Additionally, in November 2017, the Ethiopian birr was officially devalued by 15% against the US dollar, causing the exchange rate to shift from 23.3 birr per dollar to 27 birr per dollar. To provide a broader perspective, it is important to highlight the overall trend in Ethiopia’s exchange rate. For example, in 1973, the exchange rate was 2.1 birr/dollar, and by 2024, it had increased to 56.559 birr/dollar on average, demonstrating an average annual growth rate of 7.4%. ^3^^,^^8^^–^^10^

Empirical studies conducted in developing countries in general, and in Ethiopia in particular, the impact of devaluation on the balance of payments, and output growth reveals diverse perspectives and mixed results, which is positive and negative effect. Studies such as, ^6^^,^^11^^–^^18^ devaluation affects positively the balance of payment trough improving trade balance, current account balance and output growth. However, several other studies ^19^^–^^24^ suggest that devaluation negatively affect trade balance in the short run and had no any significant gain in the long run, and output is negatively affected both in the short run and short run, contractionary impact. This contractionary impact is attributed to reduced aggregate demand, income redistribution from domestic to foreign sectors, and deterioration of the trade balance. ^25^^,^^26^

Hence, this study aims to make the following contributions to the existing literature: first, while previous studies have primarily focused on examining the individual effects of devaluation on trade balance, balance of payment, and output growth; (e.g., Refs. 3, 7, 11, 13, 16, 18– 21, 27– 32), it has been observed that there are dynamic relationships among devaluation, balance of payment, and output growth. Thus, this study seeks to provide empirical evidence on the dynamics of devaluation, the balance of payment, and output growth. Second, prior studies have primarily concentrated on illustrating the transmission mechanism of monetary policy while neglecting to investigate the transmission of devaluation on the balance of payments and output growth (e.g., Refs. 4, 33, 34). Thus, further investigation is needed to explore the complex channels through which devaluation influences the balance of payments and output growth. Third, many previous studies have revealed inconclusive results regarding the dynamics of devaluation, balance of payment, and output growth. Therefore, this study contributes by providing more critical insights into these dynamics. Finally, this study resolves the devaluation puzzle by offering a more comprehensive and nuanced understanding of the transmission channels through which devaluation impacts the balance of payments and output growth. In addition to the exchange rate, this study considers other crucial variables such as foreign asset reserves, interest rates, money supply, and inflation, thereby providing a more comprehensive picture of the transmission channel. This study employs a structural VAR model and uses a comprehensive dataset covering the period from 2001Q1 to 2023Q4 to enhance our understanding of the complex dynamics between devaluation, balance of payments, and output growth in the Ethiopian context.

The remaining section of the paper is structured as follows; Section 2 presents data description and methodology, Section 3 reports results and discusses the study’s results, and Section 4 provides the conclusion and policy recommendations of the study.

## 2. Methods

### 2.1 Data

This study uses quarterly data obtained from the National Bank of Ethiopia (NBE) to investigate the effects of devaluation on the balance of payments and output. The dataset comprises seven variables: exchange rate, balance of payments, money supply, inflation, interest rates, foreign asset reserves, and real gross domestic product (GDP). To ensure consistency, the real GDP data initially available only annually from the NBE are interpolated to quarterly data using the quadratic-sum approach of E-views 12. The dataset covers a period of 92 observations from 2001Q1 to 2023Q4. The use of quarterly data facilitates the identification of structural shocks, reduces the likelihood of structural breaks, and captures important intra-year dynamics. ^35^

### 2.2 Theoretical framework and model specification

**2.2.1 Theoretical framework**

The theoretical framework connecting devaluation, balance of payments, and output originates from Keynesian macroeconomic models applied to small open economies. This relationship is essential for understanding how changes in currency value can impact overall economic performance.

To analyze this connection, we begin with the national income identity equation, which defines aggregate demand as, Y=C+I+G+X−M ; where, output (Y) is defined as the sum of consumption (C), total investment (I), government expenditure (G), and net exports (X-M) (the difference between exports and imports).

Expanding on this framework, the elasticity approach to the balance of payments analyses the impact of devaluation on a country’s current account balance, assuming perfectly elastic supply for domestic exports and foreign imports, meaning that changes in demand do not affect prices, and any shifts in relative prices result solely from changes in the nominal exchange rate. ^36^ The elasticity approach primarily focuses on the current account of the balance of payments, assessing the conditions under which exchange rate changes can offset price distortions in international trade, a key factor in the value of imports exceeding exports. ^1^ This method applies Marshallian partial equilibrium analysis to the export and import markets, assuming negligible capital movements and a domestic price level that adjusts to the world price level, with the effectiveness of devaluation in improving the balance of payments depending critically on the foreign elasticity of demand for exports and the domestic elasticity of demand for imports, denoted as e _x_ and e _m_.

If the elasticity condition holds, $e_{x}+e_{m}>1\;$ , devaluation would improve the balance of payments, assuming a stable foreign exchange market; this principle is known as the Marshall-Lerner condition. Conversely, if the sum of elasticities is less than one, devaluation negatively impacts the balance of payments, while appreciation has a positive effect, and if the sum equals one, the balance of payments remains unchanged.

There are significant concerns about the effectiveness of devaluation in developing countries, as critics argue that the elasticities of exports and imports are too low to expect an improvement in the balance of payments. ^37^^,^^38^ Additionally, skepticism arises from the lag in the current account’s response to changes in relative prices, as trade volumes tend to adjust slowly due to the inertia of importers and existing contracts, resulting in a "J Curve effect," where the balance of trade worsens before it improves.

However, the elasticity approach to the balance of payments has limitations, as it does not account for the effects of changes in export and import volumes on national income. As noted by Ref. 36, Alexander’s 1952 article critiques the elasticity approach for being a partial equilibrium analysis and advocates for the absorption approach, arguing that a favourable configuration of price elasticities alone may not yield a positive balance of payments effect from devaluation if domestic absorption is not reduced. To address this issue, the absorption approach divides national income into two components: absorption (A) and the current account (CA). Absorption is defined as, A=C+I+G , leading to the relationship CA=Y–A=X−M , which emphasizes that the current account reflects the difference between national income and domestic absorption.

The differential form of the current account equation, dCA=dY–dA , indicates that the effects of devaluation on the current account depend on its impact on national income relative to domestic absorption. As noted by Ref. 36, absorption can increase with income, influenced by the marginal propensity to absorb (a), while direct effects on absorption are represented as $A_{d}$ . For devaluation to improve the current account balance, the condition $\left(1–a\right)d\;Y>dA_{d}$ , must be satisfied, meaning that the increase in income not allocated to absorption must exceed any changes in direct absorption. Consequently, devaluation influences national income through mechanisms such as employment and terms of trade while also affecting absorption via real balance effects and income redistribution.

In monetary theory, balance of payments disequilibrium is viewed as a reflection of imbalances within the money market, consistent with the monetary approach that emphasizes the relationship between money demand and supply. This approach is based on three key assumptions: a stable money demand function, a vertical aggregate supply schedule, and the principle of purchasing power parity (PPP). These assumptions facilitate the development of a comprehensive theory of the balance of payments, where monetary flows are treated as autonomous items, while transactions related to goods, services, and investments are considered accommodating items.

The balance of payments can be represented by the equation, BOP=CA+KA+dR=0 , where dR denotes changes in central bank reserves held in foreign currency. A positive dR indicates a deficit in the combined current and capital accounts, suggesting a reduction in reserves due to the central bank’s purchase of domestic currency with foreign currency. Devaluation affects the balance of payments by influencing the demand for money in relation to its supply, as highlighted by Refs. 36, 39. The simple monetary model of the balance of payments incorporates the money demand equation derived from the quantity theory of money, represented as Md=kPy . In this equation, Md denotes the demand for nominal money balances, *p*, is the domestic price level, *y*, represents real domestic income, and *k* measures the sensitivity of money demand to changes in nominal income. This demand function is similarly applicable to foreign economies, expressed as ${M_{d}}^{*}=k^{*}P^{*}Y^{*}$ .

Under the assumption of purchasing power parity (PPP), the exchange rate SS is defined as $S=\frac{P\;}{p^{*}}$ , indicating the domestic currency value per unit of foreign currency. In equilibrium, the demand for money equals its supply in each country, leading to the relationship $\frac{\mathrm{Ms}\;}{{M_{s}}^{*}}=\frac{\mathrm{kPY}}{k^{*}P^{*}Y^{*}}$ . This relationship allows for the expression of the exchange rate as $S=\frac{\mathrm{Ms}\;}{{M_{s}}^{*}}\frac{k^{*}Y^{*}}{\mathrm{kY}}$ . Here, $M_{s}$ represents the domestic money supply, while ${M_{s}}^{*}$ denotes the foreign money supply, illustrating that the exchange rate is determined by the relative supply and demand for the respective national money stocks.

In accordance with the above theoretical framework and empirical evidence, models can be specified to capture the impact of devaluation on the balance of payments and output growth by introducing structural shocks. The theoretical model examines the transmission channels of devaluation in the balance of payments and output. The model incorporates both the demand and supply sides of the economy to analyse the effects of devaluation. ^39^ On the demand side, the model considers the national income identity equation and aggregate supply.

**The balance of payment channel:** devaluation can improve the current account of an economy in equilibrium if the combined elasticities of foreign demand for exports and domestic demand for imports exceed unity, known as the Marshall-Lerner condition. ^36^^,^^39^^,^^40^ This indicates that devaluation has both a price effect, making exports more competitive and imports more expensive, and a volume effect, leading to increased export volumes and decreased import volumes. Moreover, devaluation can impact the current account balance by influencing the marginal propensity to absorb, income levels, and direct absorption. Furthermore, the imbalance in the money market, as explained by the monetary approach, contributes to the disequilibrium in the balance of payments. In addition, shocks in the money supply, central bank’s foreign exchange reserves, inflation, and real output levels influence the balance of payments, and are specified in Eq. (1).

bop=f(reer,ms,inf,fxre,rgdp) (1)

Where, *bop* - is balance of payment, *reer* - is real effective exchange rate, *M*_*s*_ - is money supply (M2), *inf* - is inflation, *fxre* - is foreign asset reserve, and *rgdp* - is real GDP.

**The exchange rate channel: i**n a small open economy with free capital mobility, the domestic interest rate must equal the world interest rate in equilibrium. ^39^ Therefore, by equating the domestic interest rate with the world interest rate, we can determine the equilibrium level of the current account. However, in our economy, the interest rate is not determined through this mechanism but is instead fixed by the central bank. A higher domestic interest rate encourages domestic savings and reduces firms’ investment in physical capital. As a result, the domestic interest rate itself affects the current account by reducing exports. The relationship between the interest rate and the exchange rate, regardless of whether devaluation is expansionary or contractionary, is influenced by the type of shock. ^41^ This relationship between the domestic interest rate and investment demonstrates that the domestic interest rate has its own impact on the balance of payments.

This study also considers the inclusion of the price level in the balance of payment equation to account for the price environment of an economy. This is crucial in determining whether devaluation is inflationary or not, as inflation has a significant impact on output. ^42^ An inflationary environment can have negative effects on output. ^14^^,^^43^ This is because it can lead to inefficient allocation of resources due to distortions in relative prices and increased administrative costs for firms ^36^^,^^39^; clearly indicate the interaction between the balance of payments and the exchange rate. According to the elasticity approach of exchange rate determination, the exchange rate is determined by the flow of currency through the exchange rate market affecting trade balance, which affects the balance of payments component. In the portfolio view of exchange rate determination; the change in asset prices in the stock market through changes in the price of bonds and money affects the exchange rate.

Therefore, we include BOP in the exchange rate model. We have also understood that an increase in the money supply lowers interest rates, reduces borrowing costs, and promotes investment, which might enhance domestic output. In addition, a higher money supply will reduce the value of the currency. This relationship shows that money supply affects investment and output. ^41^ As in the quantity theory of money, the exchange rate is determined by the quantity of money printed by the central banks that is money supply. This aligns with the monetary approach, where both interest rates and money supply affect exchange rates. ^44^ Additionally, there is a long-run relationship between foreign reserves and exchange rates, with changes in foreign reserves leading to fluctuations in the exchange rate, but not vice versa. Based on these concepts, we can develop an exchange rate model as specified in Eq. (2):

reer=f(ms,inf,fxre,bop,rgdp) (2)

From the above theoretical framework, we can develop the model that fulfills one of the objectives of this paper, and is specified in Eq. (3):

gdp=f(reer,ms,inf,fxre,bop) (3)

The existence of exchange rate devaluation has both contractionary and expansionary effects on output through the employment effect and the terms of trade balance effects. ^36^ To show which effect will happen when the country devalues its currency; adding nominal exchange rate into the model is necessary. An increase in money supply lowers interests, reduces borrowing costs, and promotes investment which might enhance domestic output. The improvement in investment leads to increase output which intern promotes a country to export more. In addition, a higher money supply will reduce the value of currency. This relation shows money supply affects the balance of payment. ^41^ Thus, we include the money supply in the model. Table 1 summarizes the contractionary effect of devaluation on the basis of various theoretical and empirical studies, highlighting the transmission channels. Table 1 summarizes the contractionary effect of devaluation on the basis of various theoretical and empirical studies, highlighting the transmission channels.

**Table 1.** Summary of transmission channels of devaluation.

Number	Channel	Effect of devaluation
1	The balance of payment channel	When a country is initially in a trade deficit and undergoes devaluation, the value of imports surpasses that of exports. The price increase in traded goods reduces the real income of the home country, thereby raising the real income of the rest of the world.
2	Real balance channel	Devaluation results in an increase in the general price level, which leads to a decrease in the real money supply. This reduction in real money supply has the effect of reducing total expenditure and contracting output. In other words, devaluation causes prices to rise, which in turn reduces the purchasing power of money and leads to a decrease in overall spending and economic output.
3	The price channel	Devaluation of a currency results in a higher price of imported goods when measured in the domestic currency. This price increase impacts the demand for domestic products, leading to an increase in demand. Moreover, the price of domestic commodities also tends to rise. In conclusion, devaluation leads to elevated prices of imported goods in the domestic currency, which stimulates the demand for domestic products and creates upward pressure on domestic commodity prices.
5	Foreign asset reserve channel	Devaluation leads to an increase in interest rates, which in turn reduces the money supply and causes a decline in stock prices. This scenario hampers firms' ability to finance their activities, as the cost of capital rises due to higher interest rates and increased equity costs associated with issuing stocks. As a result, a decrease in investment is expected, leading to a decline in output. ^44^

**Table 2.** Variable descriptions, theoretical links, and data sources based on the theoretical framework.

Variable	Description	Theoretical Link	Data Sources
Real Gross Domestic Product (rgdp)	Total value of goods and services produced within a country, adjusted for inflation.	Measures economic performance; influenced by devaluation through employment, investment, and trade balance effects.	National Bank of Ethiopia (NBE)
Real Effective Exchange Rate (reer)	Weighted average of a country’s currency value relative to a basket of foreign currencies, adjusted for inflation, which reflects the country's external competitiveness	Devaluation lowers the REER, which can improve net exports if the sum of the price elasticities of demand for exports and imports exceeds one. A more competitive REER boosts GDP but influences inflation	National Bank of Ethiopia (NBE)
Money Supply (ms)	Broad money (M2), including cash, checking deposits, and easily convertible near money.	Increased money supply lowers interest rates, boosts investment. However, excess money supply can devalue the currency, increase inflation, and impact the balance of payments.	National Bank of Ethiopia (NBE)
Inflation (inf )	The rate at which the general price level of goods and services rises, decreasing purchasing power.	Devaluation raises domestic prices, reduces real money supply, and can contract output through cost-push inflation. High inflation reduces real money balances, discourages investment, and contracts economic output. Persistent inflation undermines competitiveness and worsens the balance of payments.	National Bank of Ethiopia (NBE)
Foreign Exchange Reserves (fxre)	External assets held by the National Bank in foreign currencies (cash, bonds, securities), used to stabilize the currency and manage external shocks.	Higher reserves stabilize the currency, while devaluation may lead to reserve depletion if central banks intervene. Reserves also influence monetary policy and reflect external sector strength.	National Bank of Ethiopia (NBE)
Balance of Payments (bop)	Record of all economic transactions between a country and the rest of the world, reflecting the country's external position	Devaluation can improve the current account if the Marshall-Lerner condition is satisfied and reduces trade deficits. Persistent imbalances may indicate structural weaknesses, requiring policy adjustments to correct deficit.	National Bank of Ethiopia (NBE)

**2.2.2 Structural Vector Autoregressive model (SVAR)**

The Structural Vector Autoregressive (here after, SVAR) model is a widely used tool for analysing the monetary transmission mechanism ^45^ and for empirically studying the impact of structural shocks empirically. ^46^^,^^47^ The SVAR model is constructed from the reduced form of the VAR ( *p*) model, which represents the data generated by the structural VAR ( *p*) model, as specified in Eq. (4);

$$B_{0}y_{t}=\alpha_{i,j}+B_{1}A_{i}y_{t‐1}+‐‐‐‐‐‐+B_{P}A_{i}y_{t‐P}$$ (4)

Where, $\alpha_{i,j}$ is an ( n×2 ) matrix constants and linear trends, $B_{i}$ , i=1,…,p, is a K×K matrix of autoregressive slope coefficients, the K×K matrix $B_{0}$ reflects the instantaneous relations among the model variables, and the K×1 vector of mean zero structural shocks $w_{t}$ is serially uncorrelated with a diagonal covariance matrix Σw of full rank such that the number of shocks coincides with the number of variables. The endogenous variable, $\;y_{t}$ , that we include the VAR is the first difference of real effective exchange rate ( $∆{\mathrm{ree}}_{t}$ ), output growth ( $∆y_{t}$ ), consumer inflation ( $∆p_{t}$ ), broad money supply ( $∆{\mathrm{ms}}_{t}$ ), foreign exchange reserve ( ∆fxre ), and balance of payment ( bop ). These variables are assumed to be a covariance stationary vector process. For ( $∆{\mathrm{ree}}_{t}$ , $∆y_{t}$ , $∆p_{t}$ , $∆{\mathrm{ms}}_{t}\;$ , $∆i_{t}$ , and ∆fxre , we can reject the hypothesis of the existence of a unit root at the 10% level (using Augmented Dickey-Fuller and Phillips-Perron tests). We can, however, not reject the hypothesis of a unit root in bop . Given the low power of these tests in relatively small data sets, we follow ^48^^–^^50^ and assume that bop is stationary since the balance of payment cannot have a unit root at level.

The reduced-form representation of the model in Eq. (5) can be obtained by pre-multiplying both sides of Eq. (4) by $\;{B_{0}}^{-1}$ , results in the model. ^46^^,^^51^^,^^52^

$$y_{t}=\lambda +A_{1}y_{t‐1}+‐‐‐‐‐‐+A_{p}y_{t‐P}+u_{t}$$ (5)

Where; $A_{i}$ = ${B_{0}}^{-1}B_{i}$ and $\;u_{t}={B_{0}}^{-1}w_{t}$ , λ = ${B_{0}}^{-1}\alpha$ and it can be estimated by maximum likelihood (ML) estimation methods.

Estimation of the matrix $B_{0}$ requires additional restrictions on the data generating process (DGP) based on economic theory. If the matrix $B_{0}\;$ can be solved for, given these restrictions and the data, we say that the structural VAR model parameters, $\left(B_{0},B_{1},\ldots ,B_{p},\mathrm{\Sigma w}\right),$ are identified or, equivalently, that the structural shocks $\mathrm{wt}=B_{0}u_{t}$ are identified. In compact form, an SVAR system relates to the following relations as specified in Eq. (6):

$$A_{0}u_{t}={\mathrm{BW}}_{t}$$ (6)

The equation (6) is known as the AB model ^53^; Where $A_{0}\;$ is (n×n) matrix of contemporaneous relations between endogenous variables, *B* is (n×n) matrix that linearly relates the SVAR residuals to the structural innovations, $u_{t}\;$ is vector of reduced-form residual, and $w_{t}\;$ is vector of structural shocks. The residual $u_{t}\;$ in the reduced form is presumed to be white noise. Therefore, we can estimate the AB model by OLS (ordinary list square).

The structural innovations $w_{t}$ can be derived from errors $u_{t}$ of the reduced form, but certain restrictions must be placed on the system. In details, $\;\frac{n\left(n-1\right)}{2}\;$ [ 1] Where n is the number of variables in the model; restrictions must be imposed on *A*0 matrix to be able to identify the structural shocks. ^54^

**2.2.3 Recursively identified structural VAR model**

In this study, based on the work of, ^46^ the response of economic variables to temporary shocks was estimated using a recursively identified structural model. This approach assumes that the current structural shocks are not influenced by the preceding ordering variables. Instead, it is assumed that the variables are affected by a sequential chain of shocks, or alternatively, the matrix $A_{0}$ is diagonal, indicating that the structural shocks are orthogonal. Specifically, the matrix $A_{0}$ takes the form of a lower triangular matrix, as illustrated below:

$$\left(\begin{array}{c} u_{t}^{\mathrm{reer}}\\ u_{t}^{\mathrm{ms}}\\ u_{t}^{\inf}\\ u_{t}^{\mathrm{fxre}}\\ u_{t}^{\mathrm{bop}}\\ u_{t}^{\mathrm{rgdp}} \end{array}\right)=\left[\begin{array}{cccccc} 1 & 0 & 0 & 0 & 0 & 0\\ b_{21} & 1 & 0 & 0 & 0 & 0\\ b_{31} & b_{32} & 1 & 0 & 0 & 0\\ b_{41} & b_{42} & b_{43} & 1 & 0 & 0\\ b_{51} & b_{52} & b_{53} & b_{54} & 1 & 0\\ b_{61} & b_{62} & b_{63} & b_{64} & b_{65} & 1 \end{array}\;\right]*\left(\begin{array}{c} w_{t}^{\mathrm{reer}}\\ w_{t}^{\mathrm{ms}}\\ w_{t}^{\inf}\\ w_{t}^{\mathrm{fxre}}\\ w_{t}^{\mathrm{bop}}\\ w_{t}^{\mathrm{rgdp}} \end{array}\right)$$

Where, ( $u_{t}^{\mathrm{reer}}$ , $u_{t}^{\mathrm{ms}}$ , $u_{t}^{\inf}$ , $u_{t}^{\mathrm{fxre}}$ , $u_{t}^{\mathrm{bop}}$ , and $u_{t}^{\mathrm{rgdp}}$ ), are the structural disturbance, that is, real effective exchange rate shocks, money supply, the price shocks, foreign asset reserve shocks, the balance of payment shocks, and output shocks respectively; and ( $w_{t}^{\mathrm{reer}}$ , $w_{t}^{\mathrm{ms}}$ , $w_{t}^{\inf}$ , $w_{t}^{\mathrm{fxre}}$ , $w_{t}^{\mathrm{bop}}$ , and $w_{t}^{\mathrm{rgdp}}$ ), are the residuals in the reduced form equations, which represent unexpected movements (given information in the system) of each variable.

In certain scenarios where a fully developed theoretical model is unavailable, the process of identification can be achieved through the incorporation of extraneous information and the selective utilization of insights from economic theory. In line with, ^46^ we present the construction of a recursive form of the SVAR (Structural Vector Autoregressive) model, as demonstrated above.

Once the structural shocks have been identified, a rigorous analysis and interpretation of these macroeconomic shocks become imperative. This analysis is conducted within the framework of structural VAR models and involves the examination of structural impulse responses as well as the implementation of forecast error variance decompositions. These analytical techniques provide valuable insights into the dynamics and impacts of the identified shocks.

## 3. Results and Discussion

### 3.1 Stationarity test

To enhance the robustness of the test, the presence of a unit root was assessed using the Augmented Dickey–Fuller (ADF) and Phillip-Perron (PP) tests. The results of the unit root tests conducted using the Augmented Dickey–Fuller and Phillip-Perron methods are summarized in Table 3. Based on the results of the ADF and PP tests, it is evident that all variables in the VAR model, except bop, are nonstationary at this level. This implies that the null hypothesis of a unit root, including both trend, and trend and intercept, cannot be rejected for these variables. However, upon considering their first differences, these variables exhibit stationarity.

**Table 3.** Augmented Dickey Fuller and Phillip-Perron (PP) unit root test.

	ADF test statistics		PP test statistics	
Variables	At level	At first difference	At level	At first difference
reer	-2.662256	-7.358477***	-1.769159	-6.670440***
lnms	-3.976900	-9.777815***	-3.921847	-12.41923***
inf	-2.438531	-4.431002***	-3.320427	-5.910959***
lnfxre	-1.226036	-9.962558***	-1.226036	-9.960837***
bop	-6.294778***	-12.43021***	-6.303920***	-26.733448***
lnrgdp	-2.338523	-9.270263***	-2.386307	-9.270263***

Source: Author’s estimation.

Note: *, **, *** represents significant at 10%, 5% and 1% level of significance respectively.

### 3.2 Lag Length and stability check

o determine the lag length of VAR/SVAR, the study employs different lag-length selection criteria, including the likelihood ratio test statistic (LR), Final Prediction Error (FPE), Akaike Information Criteria (AIC), and the Hannan-Quinn Information Criterion (HQ). In Table 4 the lag length selection criterion is tabulated. The AIC lag length section test indicates that the appropriate lag length for the VAR model is two (2).

**Table 4.** VAR lag order selection criteria.

Sample: 2001q1 thru 2023q4						Number of obs = 86		
Lag	LL	LR	df	p	FPE	AIC	HQIC	SBIC
0	-1193.62		36	0.000	52620.1	27.8981	27.967	28.0693
1	-624.186	1138.9	36	0.000	.216018	15.4927	15.9751 *	16.6913
2	-581.212	85.949	36	0.000	.185863 *	15.3305 *	16.2264	17.5565
3	-547.197	68.029	36	0.000	.200661	15.3767	16.686	18.6301
4	-511.383	71.628	36	0.000	.213805	15.381	17.1038	19.6618
5	-473.272	76.223	36	0.000	.224945	15.3319	17.4682	20.6401
6	-438.611	69.322 *	36	0.000	.27122	17.9128	21.6987	

Source: Author’s estimation.

* Optimal lag length selected by the criterion.

Prior to estimating the parameters of the SVAR model with the optimal lag length, it is essential to assess the stability conditions of the VAR model by examining the AR roots. The obtained results, which are reported in Table 5, indicate that all eigenvalues in the proposed model lie within the unit circle, signifying values that are either less than one or equal to unity. Consequently, it can be concluded that the VAR/SVAR model satisfies the requisite stability condition.

**Table 5.** Roots of characteristic polynomial (Eigenvalue stability condition).

Eigenvalue	Modulus
.9998231	.9998231
.9231988 + .02646653i	.923578
.9231988 - .02646653i	.923578
.7040893	.704089
.3997328 + .3870202i	. 556391
.3997328 - .3870202i	.556391
.4521259	.452126
-.1913816	. 191382
-.1544777 + .02950001i	.157269
-.1544777 - .02950001i	.157269

All the eigenvalues lie inside the unit circle.

VAR satisfies stability condition.

Source: Author’s estimation.

### 3.3 Co-integration test and post estimation test

The unit root test reveals that the model in our study consists of a mixture of I(1) and I(0) variables. To investigate whether these variables are cointegrated at the same order (only I(1) variables) and at different orders (I(0) and I(1)), we employ Johansen’s cointegration technique. ^36^ Our analysis is based on an SVAR model with a mixture of I(1) and I(0) variables, as described previously. ^48^^,^^55^^–^^58^ The results of Johansen’s cointegration test in Table 6 indicate the presence of multiple cointegrating equations and vectors in the case of I(1) variables, whereas for the mixture of I(0) and I(1), evidence suggests the presence of three cointegrating relationships. These findings contribute to a deeper understanding of the long-run dynamics and interdependencies among the macroeconomic variables studied.

**Table 6.** Johansen tests for cointegration.

Trend: Constant Sample: 2001q1 thru 2023q4				Number of obs = 89 Number of lags = 3	
Maximum rank	Params	LL	Eigenvalue	Trace statistic	Critical value
0	42	-654.07737	.	98.8003	94.15
1	53	-630.53324	0.40738	51.7120 *	68.52
2	62	-617.58687	0.25001	25.8193	47.21
3	69	-610.49292	0.14585	11.6314	29.68
4	74	-606.49338	0.08504	3.6323	15.41
5	77	-604.71785	0.03869	0.0812	3.76
6	78	-604.67723	0.00090		

* Selected rank.

The study investigated the presence of serial autocorrelation ( Table 7) before discussing the impulse response function. The selected lags in the VAR model exhibit no significant serial autocorrelation at the 5% level, and the parameter estimates remain stable. As a result, the SVAR models with three lags satisfy the stability condition and can be reliably estimated.

**Table 7.** Serial autocorrelation test (Lagrange-multiplier test).

lag	chi2	df	Prob > Chi2
1	42.0931	36	0.22400
2	35.0569	36	0.51328

Source: Own computation.

### 3.5 Impulse response and variance decomposition

**3.5.1 Estimation results of Impulse Response**

The impulse response functions (IRFs) derived from the Structural Vector Autoregressive (SVAR) model provide empirical evidence on the dynamic effects of real effective exchange rate (REER), money supply (M2), inflation (inf ), foreign exchange reserves (lnfxre), balance of payments (BOP), and real GDP (lnrgdp) shocks on the balance of payments in Ethiopia. These findings align with, and in some cases diverge from, the existing empirical literature on the impact of devaluation on macroeconomic variables in developing countries, particularly Ethiopia. The blue line indicates the estimated response of the BOP and output to each shock, while the red dashed lines represent the ±2 standard error (S.E.) confidence bands, reflecting the statistical uncertainty around the estimates. The x-axis represents the time horizon in quarters (ranging from 0 to 20), while the y-axis captures the magnitude of deviation from the BOP’s and output’s baseline level in response to each shock.

**The response of the balance of payment to shocks**

**Figure 1.**
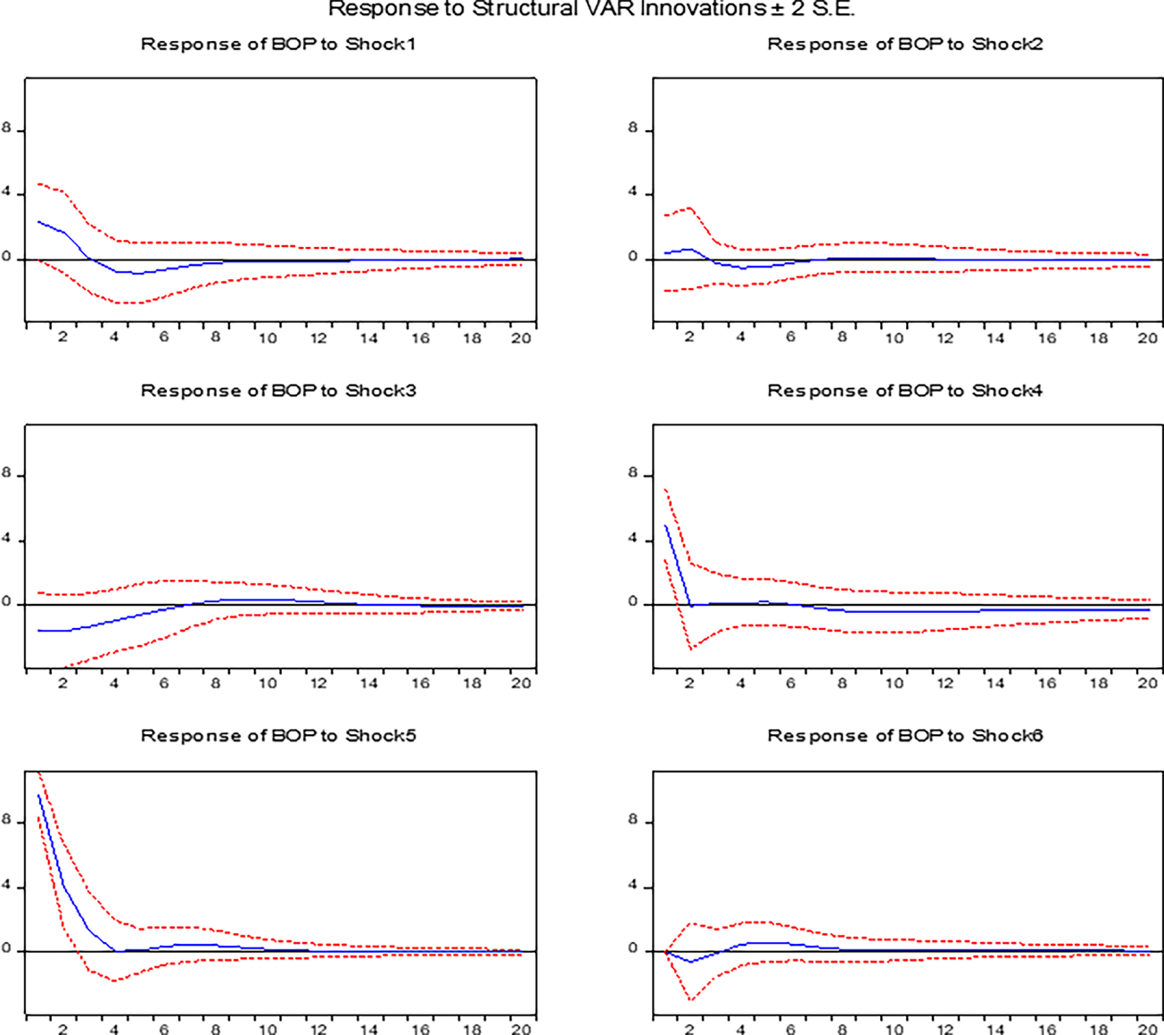
The response of the balance of payment to real effective exchange rate and other macroeconomic shocks.

Figure 1 illustrates the positive initial response of the BOP to shock 1 (REER shocks) is consistent with studies, ^6, 11– 17^ which asserts currency devaluation improves the trade balance and current account in the short run. This improvement may be attributed to increased export competitiveness and decreased import demand, consistent with the Marshall-Lerner condition. The temporary nature of the positive response reflects the initial improvement in the trade balance following currency depreciation, consistent with the findings of. ^3, 7, 16^ However, as the import costs rise and domestic prices adjust; the positive effects on the BOP gradually diminish. The BOP returns to its baseline level after approximately eight quarters, aligning with studies, ^18– 24^ which suggest that the effects of devaluation are temporary and do not yield significant long-run gains. This outcome is also consistent with the contractionary devaluation hypothesis, which posits that currency depreciation adversely affects output and the BOP in the long run by reducing aggregate demand and increasing production costs. ^25, 26^

The moderate positive response of the BOP to shock 2 (M2 shocks) aligns with the monetary approach to the balance of payments as suggested by. ^5, 33, 34^ An increase in the money supply may induce capital inflows or short-run liquidity that temporarily improves the BOP. Consistent with studies, ^18– 20^ the temporary positive effect reflects expansionary monetary policy, which may stimulate exports or reduce the real exchange rate. The declining response aligns with, ^21– 24^ which argue that in the long run, an expansionary money supply may lead to higher inflation, currency depreciation, and trade deficits, nullifying any BOP improvements.

The initial negative impact of inflation shocks (shock 3) on the BOP is consistent with studies, ^18– 24^ which highlight the contractionary effects of devaluation through rising domestic prices and imported inflation. Inflation deteriorates the BOP by increasing import costs and reducing export competitiveness, consistent with the findings of, ^25, 26^ which suggest a negative relationship between inflation and the external balance. The observed late positive effect may reflect the adjustment process where the trade balance improves as inflationary pressures are weakened by policy interventions, in line with studies. ^13, 16, 30^

The significant initial positive response of the balance of payments (BOP) to shock 4 (foreign reserve shocks) aligns with empirical evidence, ^4, 34^ which indicates that foreign exchange reserves strengthen external liquidity and mitigate the impact of external shocks. This immediate improvement corresponds with the literature suggesting that increased reserves enhance investor confidence and promote external sector stability. The subsequent decline in the BOP is consistent with studies, ^18– 24^ which argue that sustaining large reserves may require interventionist policies that disrupt trade flows and weaken external competitiveness.

The predictable positive impact of BOP shocks (shock 5) aligns with the expected dynamics outlined in studies. ^6, 11– 13^ Such shocks directly influence the external balance and reflect self-reinforcing adjustments. The immediate and sharp response aligns with prior research emphasizing the sensitivity of the BOP to external shocks. The gradual return to baseline reflects the self-correcting mechanisms in the external sector as documented by. ^16– 18^

The initial negative response of the BOP to shock 6 (real GDP shocks) reflects the standard relationship where rising domestic output increases import demand; a finding consistent with. ^25, 26^ This finding supports the argument that higher GDP growth initially worsens the BOP by increasing the consumption of imported goods. The reversal to the baseline is consistent with studies ^6, 13, 27^ that indicate sustained output growth ultimately enhances export capacity, improving the BOP.

In summary, the initial improvement in the balance of payments (BOP) following real effective exchange rate (REER) shocks highlight the short-period expansionary effects of devaluation on the trade balance. However, the eventual dissipation of BOP gains is inflationary pressures and increased import dependence limit the longer period gain of devaluation. This study offers a more comprehensive analysis of the effects of devaluation on the BOP by incorporating key variables such as money supply, foreign reserves, and inflation. Moreover, the findings highlight the dynamic relationship between devaluation, BOP, and output growth, emphasizing the short-term advantages of devaluation while recognizing its long-term trade-offs and structural limitations.

**The response of real GDP (output growth) to shocks**

**Figure 2.**
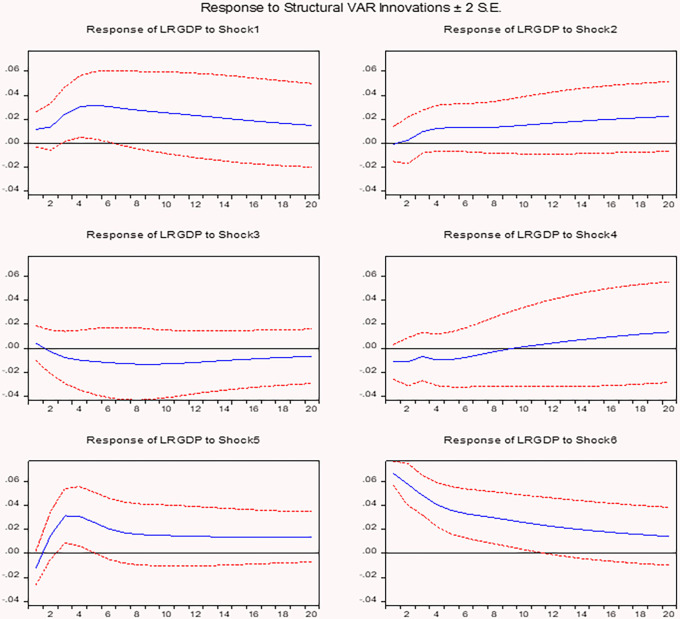
The response of output growth to real effective exchange rate and other macroeconomic shocks.

Figure 2 presents the impact of real effective exchange rate (REER) shocks (Shock 1) on real GDP (LRGDP) is positive and statistically significant during the initial periods, indicating that a depreciation or devaluation of the REER initially stimulates real GDP growth. This finding supports the argument that devaluation enhances output growth, as suggested by studies. ^6, 11– 17^ The effect remains positive but stabilizes over time, implying that devaluation may sustain output growth in the medium term (12th quarter) through increased export competitiveness and domestic production. This result is consistent with research indicating that devaluation stimulates output in the short run by enhancing net exports. ^7, 16^ However, it contrasts with studies, ^18– 24^ which propose that devaluation may have contractionary effects in the long run due to rising import costs and inflationary pressures.

Real GDP (LRGDP) exhibits a positive response to money supply shocks (Shock 2), consistent with the expansionary effects of increased liquidity on economic activity. While the impact remains positive, it gradually weakens over time, indicating that monetary expansion has a simulative effect in the short to medium term. This finding aligns with the monetary transmission mechanism, wherein an increase in the money supply enhances aggregate demand and output, as suggested by studies. ^5, 33, 34^ However, the diminishing effect over time reflects the potential for declining returns due to rising inflationary pressures, in line with the findings of. ^18– 24^

Real GDP (LRGDP) exhibits a negative and statistically significant response to inflation shocks (Shock 3), indicating that rising prices adversely affect real output. This negative impact persists throughout the observed period, suggesting that inflation imposes a sustained contractionary effect on economic growth. The findings are consistent with the contractionary devaluation hypothesis, ^25, 26^ which posits that inflation diminishes purchasing power and investment, thereby constraining output.

The response of real GDP (LRGDP) to foreign exchange reserve shocks (Shock 4) reveals a marginal positive effect, though the impact remains statistically insignificant within the confidence bands. Over time, the positive effect slightly increases, suggesting that higher reserves may contribute to economic stability and support long-term growth. This finding aligns with studies ^4, 34^ that emphasize the role of substantial foreign reserves in bolstering confidence in macroeconomic policy, thereby facilitating sustained economic expansion.

The response of real GDP (LRGDP) to balance of payments (BOP) shocks (Shock 5) exhibits a positive and statistically significant effect, indicating that improvements in the BOP are associated with higher output. This positive impact persists over time, suggesting that enhancements in the external sector contribute to sustained economic growth. These findings are consistent with studies, ^6, 13, 16^ which argue that a stronger BOP facilitates increased capital inflows and supports overall economic activity.

The positive impact of own shocks (Shock 6) on real GDP (LRGDP) gradually diminishes over time, reflecting a typical mean-reversion pattern as the economy adjusts. The effect stabilizes around zero, supporting the view that temporary fluctuations in GDP do not result in permanent changes. This finding is consistent with standard macroeconomic models, which posit that output shocks produce only transitory effects on GDP growth.

In conclusion, REER and M2 shocks positively impact GDP in the short period, however, their effects diminish over time due to inflationary pressures. Inflation shocks have a persistent negative impact, highlighting the importance of price stability. Foreign exchanges reserves provide positively contribute to long-term growth, while BOP improvements support sustained GDP growth. GDP’s own shocks exhibit a mean-reverting process, indicating temporary growth fluctuations do not lead to permanent output gains. Overall, the findings suggest that while short-term policy tools like devaluation and monetary expansion can boost economic activity, long-term growth requires maintaining price stability, strengthening the external sector, and adopting structural reforms. The impulse response analysis further reveals that REER shocks have a positive but temporary effect on money supply, inflation, and foreign exchange reserves.

Furthermore, our study reveals that positive innovations in interest rates have a negative effect on output both in the short and long run, providing empirical support for the monetary theory. This finding strengthens the existing literature by confirming the negative relationship between output and interest rates. Moreover, our analysis reveals that a positive shock in the money supply leads to a positive response in output. This observation suggests that an increase in money supply stimulates output, thereby contributing to economic growth in both the short and long run.

In line with the research conducted by ^59^ our study highlights the adverse impact of inflation on output against the findings of. ^12^ Specifically, we find that an increase in inflation significantly and negatively affects output in both the short and long run, emphasizing the detrimental consequences of rising inflation for overall economic performance. Regarding foreign asset reserves, our analysis indicates that an initial positive shock in these reserves adversely affects output in both the short and long run. However, after three quarters in the long run, the effect becomes positive, suggesting a gradual recovery and the potential for long-term benefits. This dynamic nature of the relationship between foreign asset reserves and output in the long run underscores the importance of considering the timing and persistence of shocks when assessing their effects. In addition, our study reveals a positive contemporaneous response of output to positive shocks in the balance of payments. Specifically, a surplus in the balance of payments improves the level of output, indicating a favourable impact on the economy.

In summary, the findings confirm the contractionary effect of positive shocks in the nominal exchange rate, the inverse relationship between output and interest rates, and the positive impact of money supply shocks on output. Furthermore, our study emphasizes the negative effects of inflation on output and highlights the dynamic nature of the relationship between foreign asset reserves and output.

**3.5.2 Estimation results of Variance decomposition**

The variance decomposition analysis of the balance of payment (bop) and output (lrgdp) is used to examine the strengths of various devaluation channels. The study includes forecast horizons ranging from 1 to 20 quarters. The first column of the results displays the forecast periods, while the second column represents the standard error (SE), indicating the forecast error at different quarters. Shocks denoted as Shock1 (reer), Shock2 (lnms), Shock3 (inf ), Shock4 (lnfxre), Shock5 (bop), and Shock6 (lrgdp) are used to represent each variable. Furthermore, the decomposition values for the 1st to 20th forecast horizons are presented in Tables 8 and 9.

**Table 8.** Estimation results of Variance decomposition of balance of payment.

Period	S.E.	Shock1	Shock2	Shock3	Shock4	Shock5	Shock6
1	8.050943	4.206443	0.116884	1.894437	19.64522	74.13701	0.000000
2	12.49745	5.425174	0.403668	3.300609	16.68123	73.92040	0.268927
3	15.39584	5.306358	0.433004	4.326217	16.32545	73.33826	0.270711
4	17.44638	5.585592	0.597968	4.830073	16.14733	72.43004	0.409002
5	19.06259	5.995987	0.720779	4.981017	16.00260	71.66031	0.639308
6	20.38745	6.212569	0.745677	4.981853	15.92043	71.36164	0.777833
7	21.44608	6.278243	0.743603	4.973316	15.88959	71.29066	0.824590
8	22.25370	6.287127	0.747200	5.016745	15.91322	71.20025	0.835463
9	22.84691	6.281998	0.753733	5.097787	15.97585	71.05233	0.838308
10	23.27573	6.276524	0.757284	5.175916	16.05180	70.89735	0.841128
11	23.58837	6.273777	0.757559	5.227153	16.12441	70.77090	0.846198
12	23.82267	6.272492	0.756640	5.250950	16.18785	70.67871	0.853354
13	24.00480	6.270881	0.756169	5.257565	16.24198	70.61221	0.861201
14	24.15153	6.268343	0.756790	5.256747	16.28837	70.56132	0.868431
15	24.27316	6.265205	0.758486	5.253957	16.32874	70.51920	0.874416
16	24.37600	6.261997	0.760969	5.251213	16.36441	70.48231	0.879098
17	24.46398	6.259062	0.763914	5.248888	16.39629	70.44916	0.882687
18	24.53973	6.256522	0.767071	5.246884	16.42493	70.41914	0.885449
19	24.60515	6.254368	0.770286	5.245083	16.45068	70.39196	0.887616
20	24.66176	6.252539	0.773482	5.243430	16.47378	70.36741	0.889360

**Table 9.** Estimation results of Variance decomposition of output.

Period	S.E.	Shock1	Shock2	Shock3	Shock4	Shock5	Shock6
1	8.050943	2.751871	0.011372	0.360224	2.604392	2.905382	91.36676
2	12.49745	3.632457	0.074268	0.301870	2.889086	4.203024	88.89929
3	15.39584	7.087711	0.760381	0.663543	2.330560	10.51953	78.63828
4	17.44638	10.94842	1.503362	1.086296	2.360614	13.78083	70.32048
5	19.06259	14.13262	2.104208	1.508069	2.393633	14.80717	65.05430
6	20.38745	16.48383	2.573788	1.937428	2.347995	14.82154	61.83542
7	21.44608	18.18173	2.979997	2.377590	2.221210	14.56607	59.67341
8	22.25370	19.44700	3.381576	2.808631	2.066081	14.30138	57.99533
9	22.84691	20.42981	3.813480	3.202210	1.922745	14.09408	56.53767
10	23.27573	21.21090	4.291989	3.536957	1.811425	13.94344	55.20529
11	23.58837	21.82871	4.821321	3.804809	1.740659	13.83429	53.97021
12	23.82267	22.30335	5.399420	4.008686	1.714224	13.75463	52.81969
13	24.00480	22.64968	6.021497	4.157180	1.734080	13.69777	51.73979
14	24.15153	22.88213	6.681751	4.260266	1.801045	13.65992	50.71489
15	24.27316	23.01523	7.374084	4.327034	1.914820	13.63813	49.73071
16	24.37600	23.06300	8.092429	4.364936	2.074012	13.62945	48.77617
17	24.46398	23.03832	8.830959	4.379809	2.276309	13.63091	47.84369
18	24.53973	22.95262	9.584240	4.376171	2.518700	13.63972	46.92855
19	24.60515	22.81590	10.34731	4.357557	2.797700	13.65347	46.02806
20	24.66176	22.63685	11.11571	4.326794	3.109538	13.67020	45.14091

Source: Own computation.

The variance decomposition analysis presented in Table 8 reveals the relative contributions of various shocks to fluctuations in the balance of payments (BOP) over time. In the short run, real effective exchange rate (REER) shocks (Shock 1) significantly influence BOP dynamics, second only to foreign exchange reserve shocks (Shock 4). This finding positions the foreign exchange reserve channel as a dominant factor in shaping BOP movements within the framework of exchange rate policy. Quantitatively, foreign exchange reserve shocks (shock 4) account for 19.65% of BOP fluctuations initially, with a slight decrease to 16.47% over 20 periods, indicating a stable and sustained impact. In comparison, REER shocks (shock 1) exhibit a modest but consistent effect, rising from 4.21% to 6.25% over the same period. Inflation shocks (Shock 3) gradually increase from 1.89% to 5.24%, reflecting a growing but relatively minor influence. Money supply shocks (Shock 2) remain negligible, contributing less than 1% throughout the forecast horizon. Similarly, output shocks (Shock 6) display a minimal long-term effect, starting at 0% and increasing slightly to 0.89% by the 20th period.

These results highlight the predominance of structural shocks, particularly those related to foreign exchange reserves—in explaining BOP variability, while recognizing the evolving and limited roles of other factors over time. The findings highlight the critical influence of foreign exchange reserves in maintaining BOP stability, suggesting that policy measures focusing on this channel may be more effective for long-period BOP management compared to other devaluation-related mechanisms.

The variance decomposition results of output (LRGDP) presented in Table 9 reveal the relative contribution of various shocks over a 20-period forecast horizon. The analysis indicates that REER (Shock 1) and money supply (Shock 2) shocks increase in significance, highlighting the evolving influence of external and monetary factors on output dynamics.

In the short run (Periods 1–5), output shocks (Shock 6) account for 91.37% of the variation in output during the first period, decreasing steadily to 65.05% by the fifth period. REER shocks (Shock 1) exhibit a growing influence, rising from 2.75% to 14.13%, reflecting the increasing impact of exchange rate fluctuations on output. Money supply shocks (Shock 2) also increase slightly from 0.01% to 2.10%, while inflation shocks (Shock 3) rise from 0.36% to 1.51%, indicating a limited but growing effect. Foreign exchange reserve shocks (Shock 4) remain relatively stable, contributing around 2.60% initially and remaining at a comparable level throughout this phase. BOP shocks (Shock 5) show a more substantial impact, rising from 2.91% to 14.81%, suggesting that external sector improvements contribute significantly to output changes.

In the medium term (Periods 6–12), the contribution of output shocks continues to decline, falling to 52.82% by the 12th period, indicating that the influence of endogenous output fluctuations weakens over time. Meanwhile, REER shocks increase further to 22.30%, solidifying their position as a key driver of output variation. Money supply shocks grow more pronounced, reaching 5.40%, reflecting the increasing importance of monetary policy. Inflation shocks continue to increase gradually to 4.01%, though their overall impact remains moderate. Foreign exchange reserve shocks decline slightly to 1.71%, while BOP shocks remain stable at approximately 13.75%, maintaining their role in shaping output dynamics.

In the long run (Periods 13–20), output shocks exhibit a sustained decline, accounting for only 45.14% of output variation by the 20th period, highlighting the diminishing influence of past output changes on future growth. REER shocks remain substantial, stabilizing around 22.64%–22.63%, while money supply shocks continue to grow, reaching 11.12% by the final period. Inflation shocks plateau at approximately 4.33%, while foreign exchange reserve shocks increase slightly to 3.11%, suggesting a minor but consistent contribution. BOP shocks remain relatively stable at 13.67%, indicating their persistent role in influencing long-term output variability.

Overall, the results emphasise the transmission of various shocks on output over time. While output shocks are the most dominant in the short run, their effect diminishes in the long run as REER and money supply shocks become increasingly significant. These findings suggest that exchange rate management and monetary policy play an essential role in shaping long-term output dynamics, while the influence of endogenous output factors weakens as the forecast horizon extends.

## 4. Conclusion and policy recommendations

This study addresses the devaluation puzzle by providing a more comprehensive and nuanced understanding of how devaluation affects the balance of payments and output. To achieve this, we employ a recursive structural vector autoregressive (SVAR) model focusing on Ethiopia from 2001Q1 to 2023Q4. In addition to examining the exchange rate, we incorporate other essential variables such as foreign asset reserves, interest rates, money supply, and inflation. By including these variables, we provide a more complete depiction of the transmission channel. This study also demonstrated unit root tests using the ADF and PP tests, model stability, and other necessary assessments.

The transmission of shocks on the real effective exchange rate (REER), money supply, inflation, and foreign exchange reserves (FER) significantly influence the balance of payments (BOP) and output (GDP) within an economy.

The analysis reveals that the REER is a critical determinant of the BOP. Depreciation in the REER typically enhances the BOP in the short term by improving export competitiveness and reducing import demand. This increased competitiveness can lead to heightened export volumes, thereby positively impacting GDP. However, the longer period consequences of sustained depreciation can be adverse. As import prices escalate due to a weakened currency, production costs may rise, ultimately compromising the BOP. Consequently, while the short-term effects of a depreciated currency can stimulate economic growth, long-term sustainability necessitates careful management to mitigate cost increases that could undermine competitiveness.

Similarly, the money supply has a significant influence on both the BOP and GDP. An expansion in the money supply can initially bolster the BOP by stimulating domestic consumption and investment, thereby driving economic activity. This expansionary effect tends to enhance GDP in the short run. However, if the money supply expands excessively, it may lead to inflation, eroding competitiveness and potentially contributing to trade deficits, which can harm the BOP. Therefore, although an increased money supply can support growth, it is imperative to maintain equilibrium to avoid the adverse consequences of inflation that may hinder long-term economic performance.

Inflation, as a phenomenon, generally exerts a negative impact on both the BOP and GDP. Rising prices raise import costs and diminish export competitiveness, adversely affecting the BOP. In the short term, inflation can deter investment and reduce consumption, resulting in a negative impact on GDP growth. Over the long term, persistent inflation can create structural imbalances within the economy, further constraining growth by diminishing real income and purchasing power. Consequently, effective control of inflation is paramount for maintaining economic stability and fostering sustainable growth.

Foreign exchange reserves (FER) represent another crucial factor influencing the BOP and output. An increase in foreign reserves strengthens external liquidity and enhances investor confidence, which can improve the BOP in the short term. Adequate reserves provide a buffer to manage external shocks, thereby supporting currency stability and fostering an environment conducive to investment and economic growth. In the long run, the maintenance of sufficient foreign reserves is essential for ensuring economic stability and fulfilling external obligations, which contributes positively to GDP growth.

The interconnectedness of these factors engenders a complex dynamic in economic management. For example, an increase in the money supply may catalyse inflation, which can adversely affect both the BOP and GDP. Similarly, while a depreciated REER may stimulate output in the short term, it may simultaneously induce inflationary pressures that compromise long-term growth. Adequate foreign exchange reserves can mitigate some of the negative effects stemming from inflation and currency fluctuations, thereby supporting both BOP and GDP growth. An understanding of these relationships is crucial for policymakers tasked with devising effective economic strategies that harmonize short-term simulative measures with long-term objectives of economic stability.

In summary, our analysis indicates that to foster sustainable economic growth in Ethiopia, policymakers should prioritize enhancing foreign reserve management to ensure liquidity and stability in the balance of payments, alongside implementing effective inflation control measures. Furthermore, adopting a balanced exchange rate policy is crucial to mitigating the adverse effects of prolonged currency devaluation. Continuous monitoring and evaluation of these policies will facilitate timely adjustments to emerging challenges, ultimately enhancing the country’s competitiveness in the global market.

## Data Availability

Zenodo: Unravelling the Devaluation Puzzle: Empirical Insights into the Transmission Channel on Balance of Payments and Output in Ethiopia https://doi.org/10.5281/zenodo.14910637. ^60^ This project contains the following underlying data:
-
data1.xlsx

## Footnotes

^1^
The contemporaneous matrix requires $\left(\frac{6\left(6-1\right)}{2}=15\right)$ restrictions for exact identification more than 15 restrictions were imposed, leading to over identification *.*

## Software availability

Available from: https://www.google.com/url?sa=t&source=web&rct=j&opi=89978449&url=https://www.stata.com/stata17/&ved=2ahUKEwi03vfG_pGGAxUzgv0HHQ7yC2sQFnoECC4QAQ&usg=AOvVaw03lYw5vn_Q1Y0pZIr3mJKg

License: ©Copyright 1996–2024 StataCorp LLC

https://www.google.com/url?sa=t&source=web&rct=j&opi=89978449&url=https://www.eviews.com/download/ student12/&ved=2ahUKEwjfiguqwJnl_pGGAxVLg_0HHToUAGIQjBB6BAgaEAE&usg=AOvVaw30-y1H1GZcVbJaDDX-SPiz

License: ©Copyright 2022 EViews, IHS Global Inc.
