# Supplementary material for: Unravelling the devaluation puzzle: Empirical insights into the transmission channel on balance of payments and output in Ethiopia
Source: F1000Res. 2025 May 7;13:685. Originally published 2024 Jun 24. [Version 2] doi: 10.12688/f1000research.151984.2 (PMC12166354; doi:10.12688/f1000research.151984.2)
Supplement: Peer review history

# Reviewer report: Reviewer response for version 2

**DOI:** 10.5256/f1000research.178608.r383669

**Published:** 2025-06-13

**Authors:** Zhandos Ybrayev

**Affiliations:** 1 National Bank of Kazakhstan, Kazakhstan, Kazakhstan

I have checked the amendments from version 1, and read the revised version. I believe that all necessary changes were made, and the methodology and empirical tests are valid for the hypothesis authors aim to tackle. 

 One quick comment is that money supply is in the nominal terms, while all other variables expressed in real terms (REER, RGDP, FXRE). Although, I believe that results will not be affected much, I would have recommended to have all the data series in real terms.

Is the work clearly and accurately presented and does it cite the current literature?

Yes

If applicable, is the statistical analysis and its interpretation appropriate?

Yes

Are all the source data underlying the results available to ensure full reproducibility?

Yes

Is the study design appropriate and is the work technically sound?

Yes

Are the conclusions drawn adequately supported by the results?

Yes

Are sufficient details of methods and analysis provided to allow replication by others?

Yes

Reviewer Expertise:

macroeconomic theory

I confirm that I have read this submission and believe that I have an appropriate level of expertise to confirm that it is of an acceptable scientific standard, however I have significant reservations, as outlined above.

# Reviewer report: Reviewer response for version 2

**DOI:** 10.5256/f1000research.178608.r383485

**Published:** 2025-06-09

**Authors:** Michael Connolly

**Affiliations:** 1 University of Miami, Coral Gables, Florida, USA

This is a substantial improvement, using the REER instead of the nominal exchange rate, and adding the absorption approach to the elasticity approach to the change in the exchange rate.  I recommend favorably to its indexing in its revised version.

Is the work clearly and accurately presented and does it cite the current literature?

Yes

If applicable, is the statistical analysis and its interpretation appropriate?

Partly

Are all the source data underlying the results available to ensure full reproducibility?

Partly

Is the study design appropriate and is the work technically sound?

Partly

Are the conclusions drawn adequately supported by the results?

Partly

Are sufficient details of methods and analysis provided to allow replication by others?

Yes

Reviewer Expertise:

NA

I confirm that I have read this submission and believe that I have an appropriate level of expertise to confirm that it is of an acceptable scientific standard.

# Reviewer report: Reviewer response for version 1

**DOI:** 10.5256/f1000research.166683.r305323

**Published:** 2024-09-28

**Authors:** Florencia Médici

**Affiliations:** 1 Consejo Nacional de Investigaciones Científicas y Técnicas, Buenos Aires, Argentina

The aim of the paper is to empirically study the effects of currency devaluation on output and the balance of payments (BoP), considering other relevant macroeconomic variables.

 *p.4 The authors: “ *By employing rigorous econometric techniques and a sound theoretical framework, this study provides valuable insights into the dynamics of exchange rate transmission in relation to the Ethiopian economy…. The model specification begins with the identification and determination of independent variables based on economic theory*.”

 From my point of view, the paper presents both theoretical and empirical issues that require further attention.

 Firstly, the theoretical framework needs to be more clearly articulated. While I assume the authors adopt a New Keynesian approach, the transmission mechanisms of the variables of interest are not explicitly defined, nor is it clear which variables are treated as independent.

 One key omission is the role of international financial flows, which is crucial when discussing balance of payments in underdeveloped countries. I recommend the authors review the relevant literature on this topic to address potential omitted effects, such as the interaction between interest rates, financial flows, and the real exchange rate. For instance, an increase in the domestic interest rate may attract capital inflows, resulting in a short-run balance of payments surplus and currency appreciation. This could potentially reduce inflation, improve real wages, and ultimately have a positive impact on GDP.

 *p.3 “ *In addition, devaluation does not have a significant impact on Ethiopian economy because it increases the rate of growth of imports and decreased the rate of growth of export*.”

 What is the mechanism behind this assertion? Devaluation typically has a contractionary effect, at least in the short run, which would generally lead to a reduction in imports.

 laiming that *devaluation "increases* the rate of growth of imports and decreases the rate of growth of exports" seems to imply that the country in question set international prices—a challenging assumption for a developing country. This reasoning needs to be supported by a clear explanation of the transmission mechanism at play. (Also, please refer to transmission channel 1 in Table 1 for further clarification.)

 *p.4 The authors consider the money supply to be exogenous (determined by the central bank), making the interest rate endogenous. However, both variables are included in the VAR as independent variables. While this may be theoretically acceptable, it creates ambiguity in the model structure. If the money supply is exogenous, the interest rate should be excluded from the set of independent variables, or a new equation should be added to represent the determination of the money supply (M). Alternatively, the model should account for endogeneity by employing appropriate techniques to address this issue.

 Moreover, assuming that money is exogenous while the interest rate is endogenous requires a more robust theoretical discussion to support this assumption.

 *p.4 “ *This is seeing the exchange rate from the viewpoint of the monetary approach; interest rate and money supply influence exchange rate determination. There is a long run relationship between foreign reserves and exchange rate. Moreover; any changes in foreign reserves would lead to fluctuations in the exchange rate but not vice versa.*”

 Foreign reserves are a stock variable derived from flow variables, specifically the outcome of the balance of payments (BoP). If the exchange rate affects the BoP, it is inconsistent to assume that variations in the nominal exchange rate (NER) do not influence foreign reserves. This concept needs to be reconsidered in light of the interdependencies between these variables.

 So far, the theoretical framework is not well articulated, making the transmission mechanisms confusing. This, in turn, limits the specification of the empirical model

**EMPIRICAL EVIDENCE**

- The inclusion of both the interest rate (i) and money supply (Ms) as independent variables, as mentioned earlier, raises some concerns. These variables need to be more carefully justified within the model.
- I recommend providing clearer definitions of the variables used, along with the sources from which this data was collected. This will enhance the transparency and replicability of the study.

**Unit Root Tests**:

- Clarification is needed on the variable “inf.” Is this inflation measured as consumer price variation? Since "inf" is presented as the difference of the price variable, this suggests that prices might be treated as an I(2) variable. If this is the case, further explanation is necessary.
- It would also be helpful if Table 2 included information on the significance of the trend and constants for each variable. This is important for understanding the behavior of the variables and for assessing the stationarity assumptions used in the model.

**Cointegration**:

- In Table 5, the results indicate the presence of 2 cointegration relationships, not 3. This discrepancy should be addressed for accuracy.
- On p.9, the authors state, “These findings contribute to a deeper understanding of the long-run dynamics and interdependencies among the macroeconomic variables studied.” However, it is unclear what specific dynamics are revealed by the cointegration test. A more detailed explanation of the nature of these long-run relationships would strengthen the paper's contributions.
- Additionally, please note that if "Ms" (money supply) and "BoP" (balance of payments) are removed from the set of independent variables, as previously suggested, the cointegration relationship may no longer hold. This could significantly alter the interpretation of the long-run dynamics and should be carefully reconsidered.

**Impulse Response and Variance Decomposition**:

- By definition, a VAR model is used to analyze short-run relationships. How do the authors define the “long run” in this context? Typically, in econometric analysis, when persistent relationships between variables (i.e., long-run relationships) are present, a Vector Error Correction (VEC) model is employed. The VAR model, on the other hand, is generally built with I(0) stationary variables that do not exhibit long-run relationships.
- Regarding the impulse response functions, it is unclear what specific shock each graph in Figures 1 and 2 represents, making it difficult to follow the results. Additionally, many of these results indicate no significant relationships, even in the short run, as the impulse response functions—including the confidence intervals—hover around zero.

**Conclusion**:

 In conclusion, while the empirical study on the macroeconomic effects of exchange rates is relevant and timely, the paper requires significant revisions to improve its consistency, both in the theoretical framework and the development of the empirical section.

 Arestis P. 2009. Ref 1 

 Bahmani M. H. Harvey and S. W. Hegerty. 2013. Ref 2

 Borio C. and P. Disyatat. 2015.Ref 3

 Kohler K. 2022. ‘Ref 4 

 Krugman, P., & Taylor, L. (1978). Ref 5

 Lavoie, M. (2014).Ref 6

 Reinhart C. 1995. ‘Ref 7

 Rey H. 2015. ‘Ref 8

 Rey H. 2016. Ref 9

 Serrano, F., & Summa, R. (2015) Ref 10

 Serrano, F., Summa, R., & Aidar, G. (2021). Ref 11

Is the work clearly and accurately presented and does it cite the current literature?

Partly

If applicable, is the statistical analysis and its interpretation appropriate?

Partly

Are all the source data underlying the results available to ensure full reproducibility?

Yes

Is the study design appropriate and is the work technically sound?

Partly

Are the conclusions drawn adequately supported by the results?

Partly

Are sufficient details of methods and analysis provided to allow replication by others?

Partly

Reviewer Expertise:

Macroeconomics

I confirm that I have read this submission and believe that I have an appropriate level of expertise to confirm that it is of an acceptable scientific standard, however I have significant reservations, as outlined above.

## References

[1] : Holocene ecosystem and temperature development inferred from invertebrate remains in Zminje Jezero (Dinaric Alps, Montenegro). *J Paleolimnol* .2024;72(3) : 10.1007/s10933-024-00334-y 343-361 10.1007/s10933-024-00334-y 39329145 PMC11422249

[2] : Empirical tests of the Marshall‐Lerner condition: a literature review. *Journal of Economic Studies* .2013;40(3) : 10.1108/01443581311283989 411-443 10.1108/01443581311283989

[3] : Capital flows and the current account: Taking financing (more) seriously. *Research gate* .2015;

[4] : Capital flows and geographically uneven economic dynamics: A monetary perspective. *Environment and Planning A: Economy and Space* .2022;54(8) : 10.1177/0308518X221120823 1510-1531 10.1177/0308518X221120823

[5] : Contractionary effects of devaluation. *Journal of International Economics* .1978;8(3) : 10.1016/0022-1996(78)90007-7 445-456 10.1016/0022-1996(78)90007-7

[6] : Marc Lavoie, Post-Keynesian Economics: New Foundations. *Revue de la régulation* .2014;16: 10.4000/regulation.10921 10.4000/regulation.10921

[7] : Devaluation, Relative Prices, and International Trade: Evidence from Developing Countries. *JSTOR* .1995;290-312

[8] : Dilemma not Trilemma: The Global Financial Cycle and Monetary Policy Independence.2015; 10.3386/w21162 10.3386/w21162

[9] : International Channels of Transmission of Monetary Policy and the Mundellian Trilemma. *IMF Economic Review* .2016;64(1) : 10.1057/imfer.2016.4 6-35 10.1057/imfer.2016.4

[10] : Mundell–Fleming without the LM curve: the exogenous interest rate in an open economy. *Review of Keynesian Economics* .2015;3(2) : 10.4337/roke.2015.02.08 248-268 10.4337/roke.2015.02.08

[11] : Exogenous interest rate and exchange rate dynamics under elastic expectations. *Research gate* .2021; 10.13140/RG.2.2.34473.11364 10.13140/RG.2.2.34473.11364

[12] : Devaluation, Relative Prices, and International Trade: Evidence from Developing Countries. *Staff Papers - International Monetary Fund* .1995;42(2) : 10.2307/3867574 10.2307/3867574

# Reviewer report: Reviewer response for version 1

**DOI:** 10.5256/f1000research.166683.r297002

**Published:** 2024-07-19

**Authors:** Michael Connolly

**Affiliations:** 1 University of Miami, Coral Gables, Florida, USA

Firstly, it is hard to think of the nominal exchange rate as an exogeneous variable, though in Ethiopia there seem to have been large devaluations. The issue is that under fixed or crawling exchange rates, the authorities often sell reserves to defend the exchange rate while the price of non-traded goods rises due to money base expansion to finance the fiscal deficit. So, the devaluations may be "catch up" to an overvaluation.  Indeed, they may get closer to the equilibrium where M/E, M/P, and interest rates are in equilibrium (though with expected devaluation and possibly default, there are risk premia).

 Dear The exercise has some interest though since it reaches sensible long run results. The article is too long winded and this SVAR methodology crunches out all sorts of stuff.  When the authors say exchange rate shocks negatively affect foreign exchange reserves in the short and long run, they presumably mean devaluations, since a shock could also be an appreciation. The exposition is a bit sloppy. Reliance on the Marshall Lerner condition is an old partial equilibrium elasticity result. The table is interesting and the real cash balance effect of Alexander is mentioned. I like the table, but in light of all these channels and not that many degrees of freedom, it would behoove the authors not to overinterpret causation and results. In any case, as in Mundell's analysis you either set the money supply and the exchange rate adjusts or you set the exchange rate and the money supply adjusts (unless you have exchange controls). So this is sort of a chicken and egg issue, is the exchange rate the control variable or is the money supply the control variable.

 Still, I found the results interesting and would consider a revision. However the authors have submitted 8 tables and 5 figures (each containing 7 impulse figures for a total of 35 figures altogether)  T In theory, money is neutral, the exchange rate is neutral, departing from an equilibrium. Real variables should not be affected for unanticipated one time shocks. In the case of Ethiopia, though the expectations of devaluation can change much both before and after the devaluation. 

 The authors do not have clean cut tests in my view, nor do they have clear causations. If they found no major long run changes to real variables, that would be interesting. But money or the exchange rate should be the long run determinant of nominal variables. Table 1 is good, but in any case, if the authors resubmit, I would constrain them to two sets of tables, and two sets of figures. The other results can be explained in text form, if necessary.

 At the end of the day, I feel the authors claim too much. The model  obviously does not start in equilibrium nor reach equilibrium in the short or even long run. Further, it is sort of a kitchen sink model, several factors working in different directions at the same time on the dependent variables. In statistical analysis, the idea is to test hypotheses where an exogeneous variable changes and the hypothesized change is not rejected. The model could instead have had the exchange rate as the dependent variable and the money supply as the independent variable.

 So this could be treated as such and if the long run outcomes are not significant this would not reject the hypothesis that changes in nominal variable do not affect long run real variables. That would be useful and less speculative. The authors would not need to report every single page of the SVAR program. Have fewer objectives that can be analyzed. Unravelling the details of the channels is not done nor can it be done with these techniques. I would suggest a less enticing title.

Is the work clearly and accurately presented and does it cite the current literature?

Yes

If applicable, is the statistical analysis and its interpretation appropriate?

Partly

Are all the source data underlying the results available to ensure full reproducibility?

Partly

Is the study design appropriate and is the work technically sound?

Partly

Are the conclusions drawn adequately supported by the results?

Partly

Are sufficient details of methods and analysis provided to allow replication by others?

Yes

Reviewer Expertise:

NA

I confirm that I have read this submission and believe that I have an appropriate level of expertise to confirm that it is of an acceptable scientific standard, however I have significant reservations, as outlined above.
